# Supplementary material for: Robustly detecting differential expression in RNA sequencing data using observation weights
Source: Nucleic Acids Res. 2014 Apr 20;42(11):e91. doi: 10.1093/nar/gku310 (PMC4066750; doi:10.1093/nar/gku310)
Supplement: SUPPLEMENTARY DATA [file supp_42_11_e91__index.html]

Robustly detecting differential expression in RNA sequencing data using observation weights — Robustly detecting differential expression in RNA sequencing data using observation weights — SUPPLEMENTARY DATA 

# Robustly detecting differential expression in RNA sequencing data using observation weights

## SUPPLEMENTARY DATA

**Files in this Data Supplement:**

- SUPPLEMENTARY DATA
- SUPPLEMENTARY DATA
- SUPPLEMENTARY DATA
