# Supplementary material for: Robustly detecting differential expression in RNA sequencing data using observation weights
Source: Nucleic Acids Res. 2014 Apr 20;42(11):e91. doi: 10.1093/nar/gku310 (PMC4066750; doi:10.1093/nar/gku310)
Supplement: SUPPLEMENTARY DATA [file supp_gku310_nar-03519-met-n-2013-File002.pdf]

Supplementary Data for "Robustly detecting differential expression in RNA sequencing data using observation weights"

Xiaobei Zhou, Helen Lindsay and Mark D. Robinson

March 10, 2014

Contents

1 Supplementary Figure **1**

2 Rcode **18**

2.1 Rcode for Supplementary Figure 18

2.2 Rcode for Supplementary Table 43

List of Supplementary Figures

1 Simulation keeping top dispersion 1

2 Zero inflation estimation for Pickrell dataset 2

3 Convergence of iterative estimated dispersion of ***edgeR-robust*** 3

4 Validation of simulation model 4

5 Cumulative distributions of weights 5

6 log2 of the ratio of outlier to non-outlier CPM 6

7 The effect of outliers on dispersion estimation by "pooled" and "pooled-CR" method in ***DESeq*** 7

8 Outlier detection of ***edgeR-robust*** and ***DESeq2*** 8

9 Turn off outlier detection in ***DESeq2*** 9

10 Low outlier performance of ***edgeR-robust for Pickrell data*** 10

11i Simulation based on Pickrell dataset: nTags=30000, foldDiff=2, group = 5vs5 , pOutlier=0.1, outlierMech=S, pDiff=0.1 11

11ii Simulation based on Pickrell dataset: nTags=30000, foldDiff=3, group = 5vs5 , pOutlier=0.1, outlierMech=S, pDiff=0.1 12

11iii Simulation based on Pickrell dataset: nTags=30000, foldDiff=6, group = 5vs5 , pOutlier=0.1, outlierMech=S, pDiff=0.1 13

11iv Simulation based on Pickrell dataset: nTags=30000, foldDiff=2, group = 3vs3 , pOutlier=0.1, outlierMech=S, pDiff=0.1 14

11v Simulation based on Pickrell dataset: nTags=30000, foldDiff=3, group = 3vs3 , pOutlier=0.1, outlierMech=S, pDiff=0.1 15

11vi Simulation based on Pickrell dataset: nTags=30000, foldDiff=6, group = 3vs3 , pOutlier=0.1, outlierMech=S, pDiff=0.1 16

12i 5 simulations for each setting based on Pickrell dataset: group=10v10, nTags=10000 17
12ii 5 simulations for each setting based on Pickrell dataset: group=3v3, nTags=10000 . 17

1 Supplementary Figure

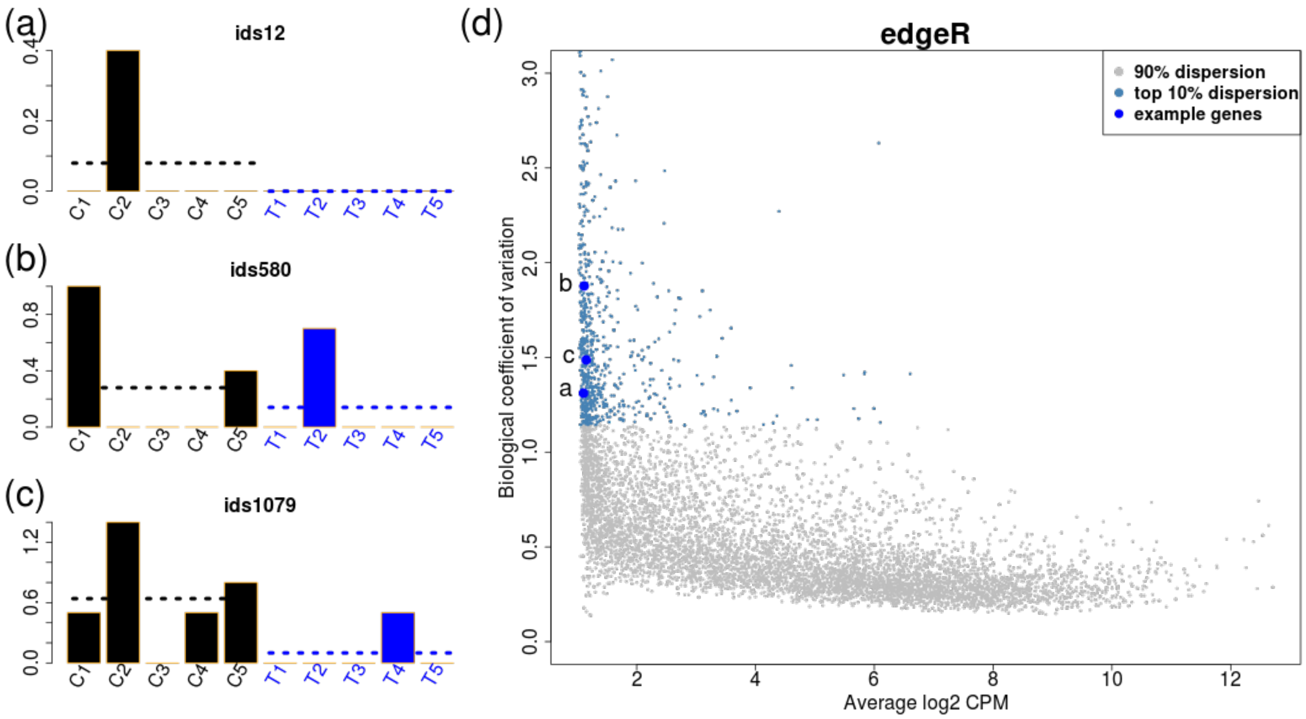

Supplementary Figure 1: Simulation keeping top 10% dispersion based on Pickrell dataset. (a), (b) and (c) show barplots of three genes from the top 10% dispersion. (d) plots genewise biological coefficient of variation (BCV) against gene abundance (in log2 counts per million) for ***edgeR***. Steel blue dots shows top 10% dispersion and 3 selected genes are labeled with large blue dots.

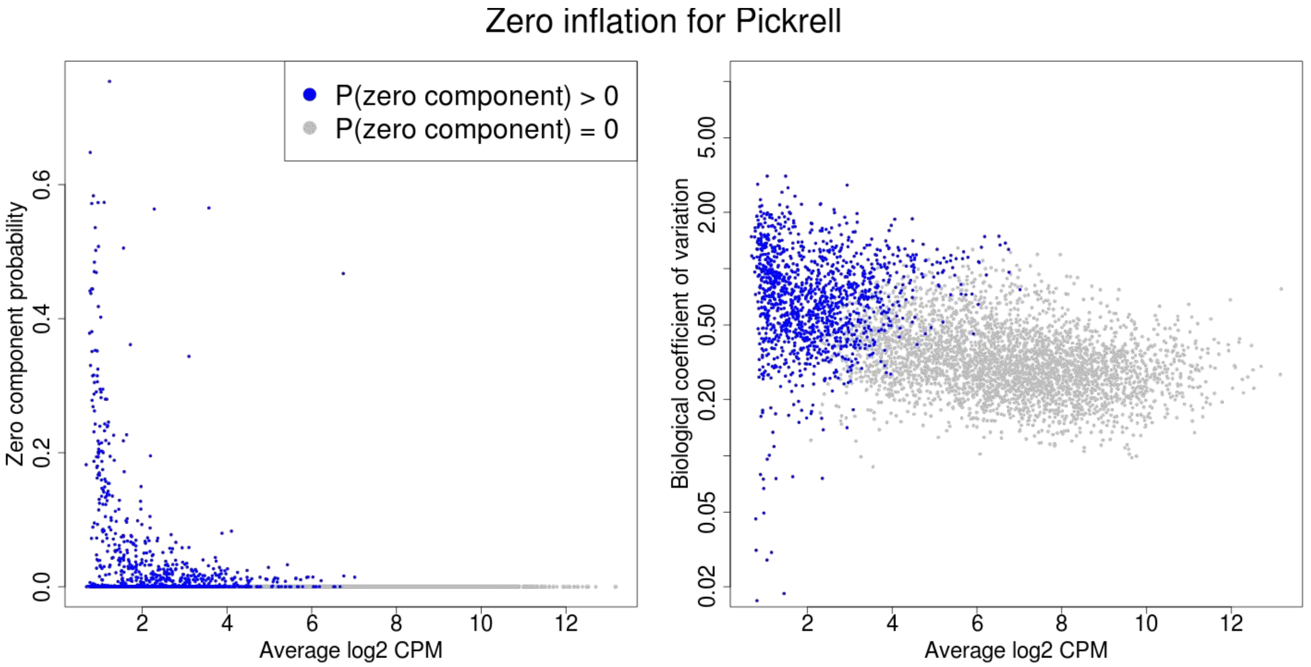

Supplementary Figure 2: Zero inflation estimation for the Pickrell dataset. We wanted to determine whether the dispersion-mean trend can be explained by zero inflation in the negative binomial model (ZINB). The left panel shows the zero component probability, $Pr(y_{gi} = 0)$, versus gene abundance (in log2 counts per million). The right panel shows feature-wise biological coefficient of variation (BCV) estimates against expression strength and highlights the zero-inflation does not account for all features with positive zero component colored blue; remaining genes are colored gray. We used the ***pscl*** R package to estimate zero-inflated negative binomial estimates [1].

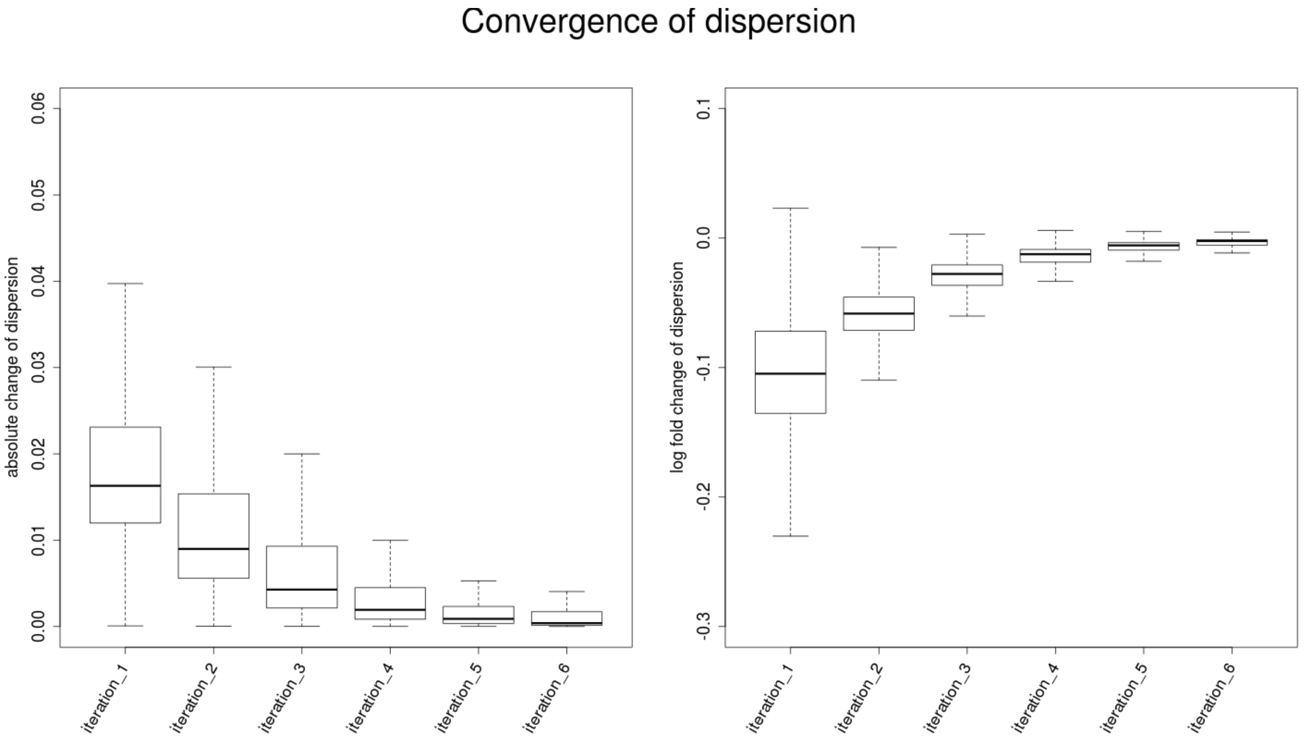

Supplementary Figure 3: Convergence of iterative estimated dispersion of ***edgeR-robust*** for the Pickrell dataset. The boxplot of absolute change (x+1 minus x) of tagwise dispersion (left). The boxplot of fold change (x+1/ x) of tagwise dispersion (right).

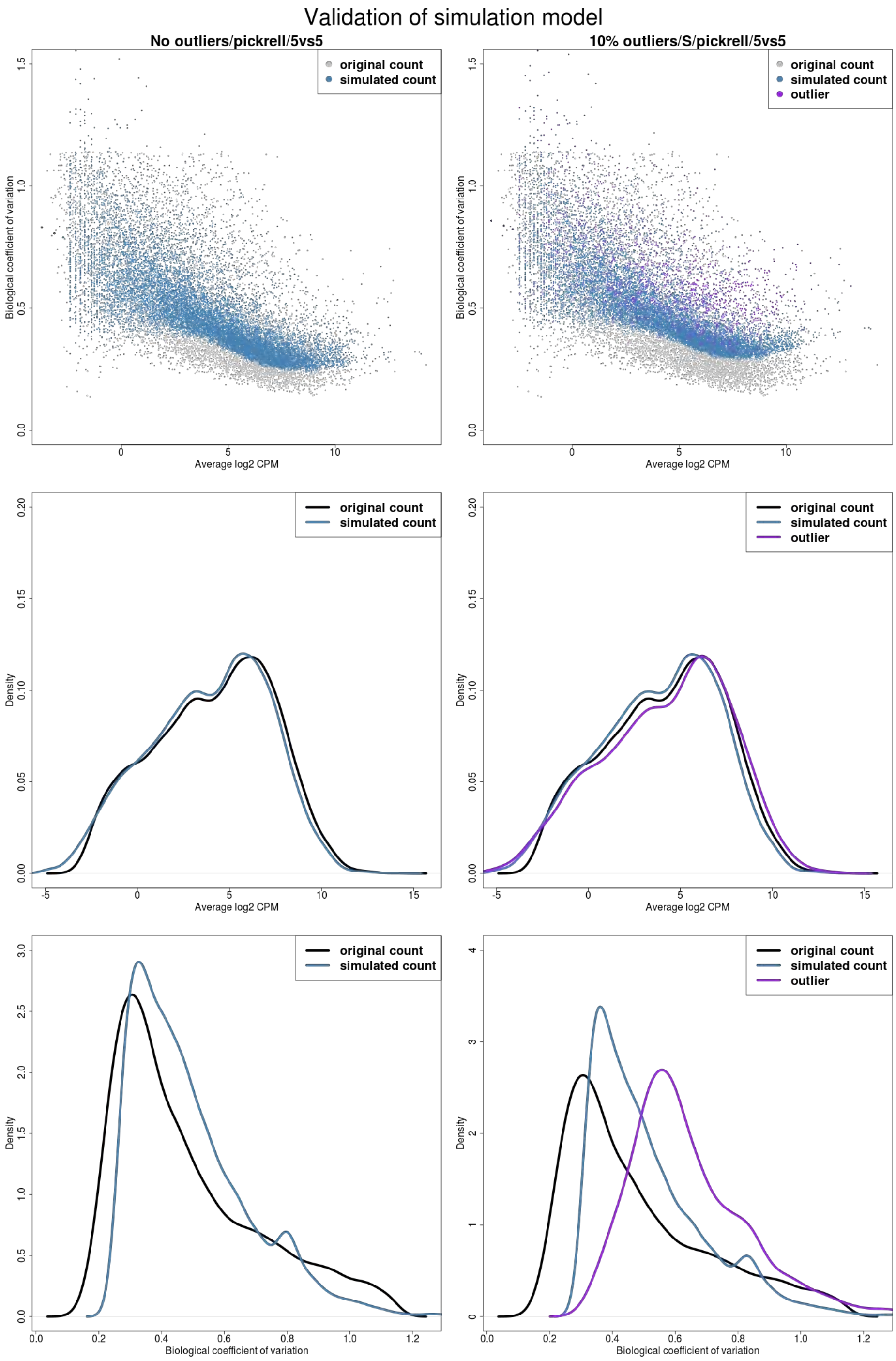

Supplementary Figure 4: Validation of simulation model. The left panels show estimates or distributions from the original counts (Pickrell dataset) as well as simulated counts based on the estimates from the dataset; the right panels show the same with 10% outliers added. (top) Feature-wise BCV against expression strength; (middle) marginal density of expression strength; (bottom) marginal density of dispersion estimates.

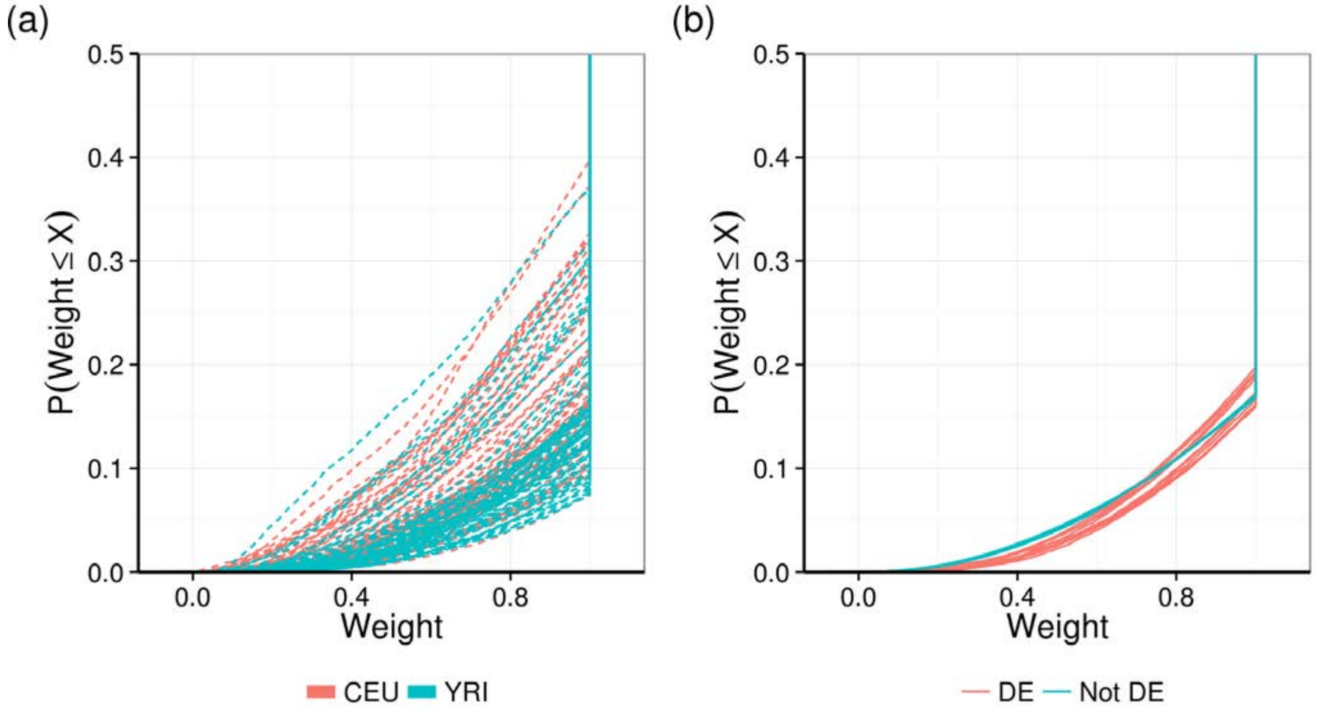

Supplementary Figure 5: Cumulative distributions of weights for (a) all samples from the Montgomery/Pickrell data set and (b) random sets of 10 samples from each population Cumulative distributions were calculated separately for differentially expressed (DE) and non-differentially expressed (Not DE) genes, where ***SAMseq*** [2] was used to test for differential expression. Note that the y-axes have been truncated at 0.5 for visibility.

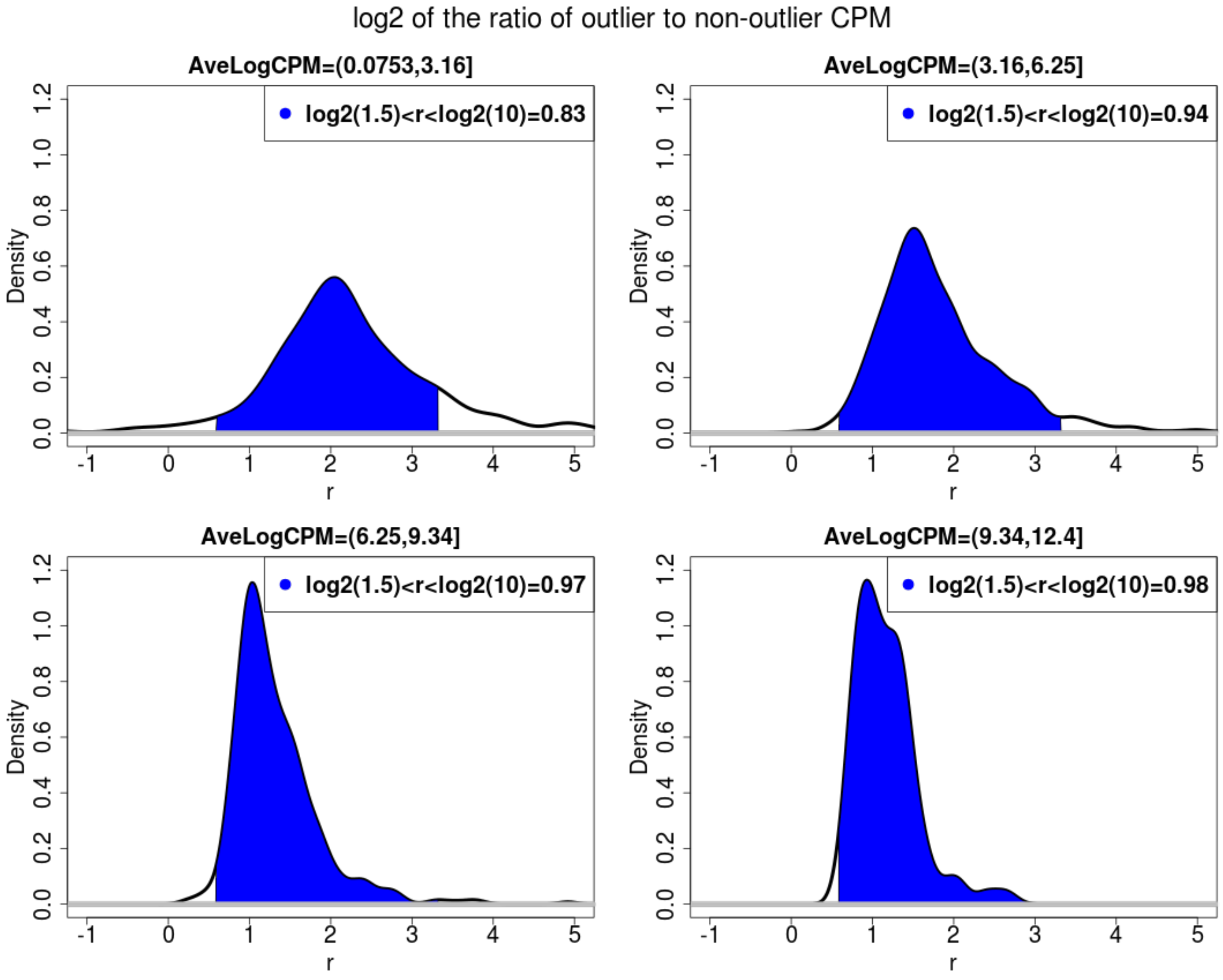

Supplementary Figure 6: log2 of the ratio of outlier to non-outlier CPM. The density of (log2) ratios of outlier (containing one observation weight less than 0.5) to non-outlier CPM is shown, split across four equally-sized average-log-CPM groups from the original counts (Pickrell dataset). The cumulative probability of ratios between 1.5 and 10 (blue) is calculated. ***Note*** that the outlier factor in the simulation is randomly chosen from this range (1.5-10).

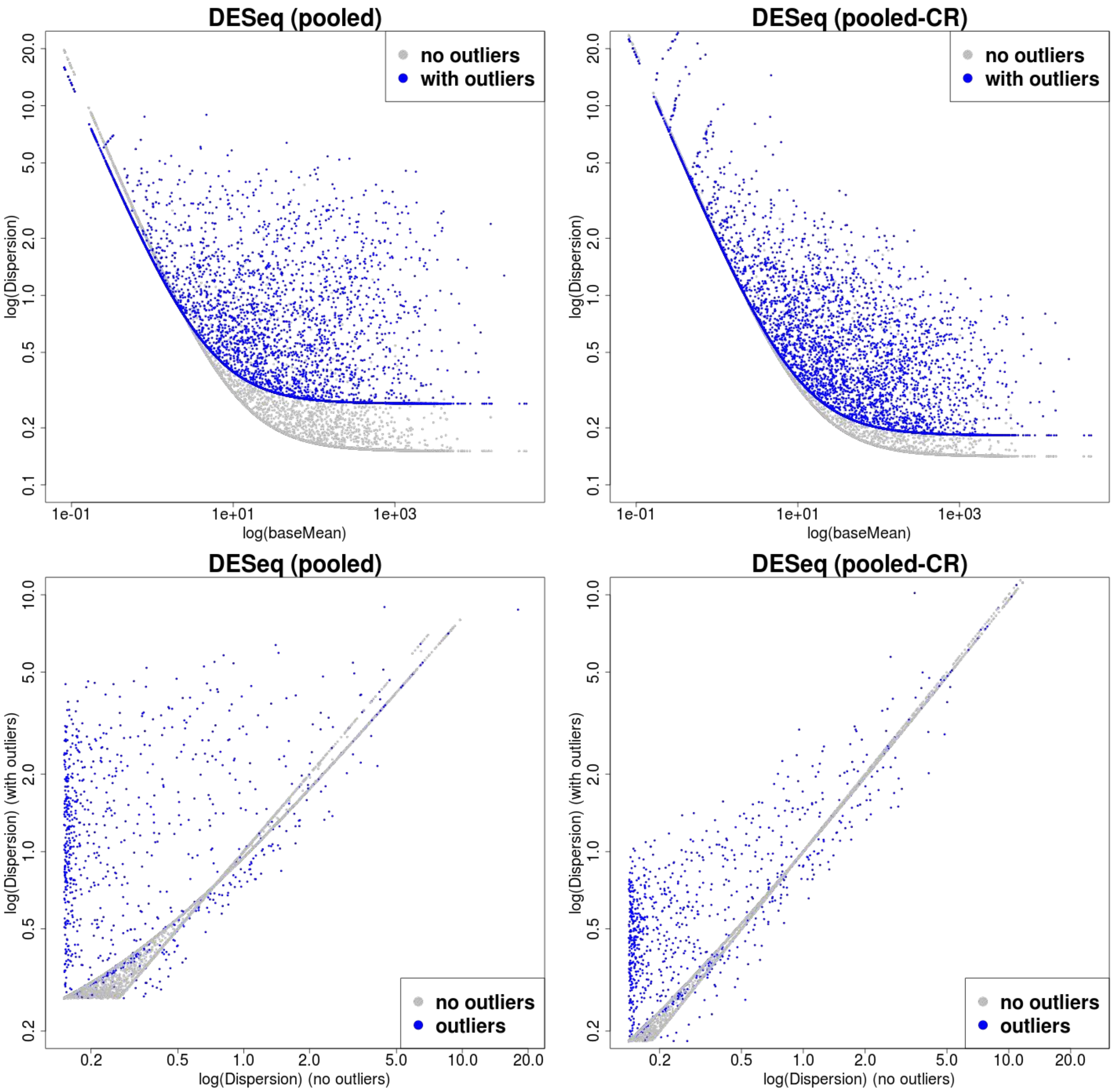

Supplementary Figure 7: The effect of outliers on dispersion estimation by "pooled" and "pooled-CR" method in ***DESeq***. Here, we show the global effect on dispersion estimation in ***DESeq*** by adding 10% outliers (i.e., 10% of observations have a single outlier), according to the "pooled" and "pooled-CR" settings. (top) feature-wise BCV against expression strength. (bottom) scatter plot of (log) (dispersion) between simulated counts with outliers and without outliers.

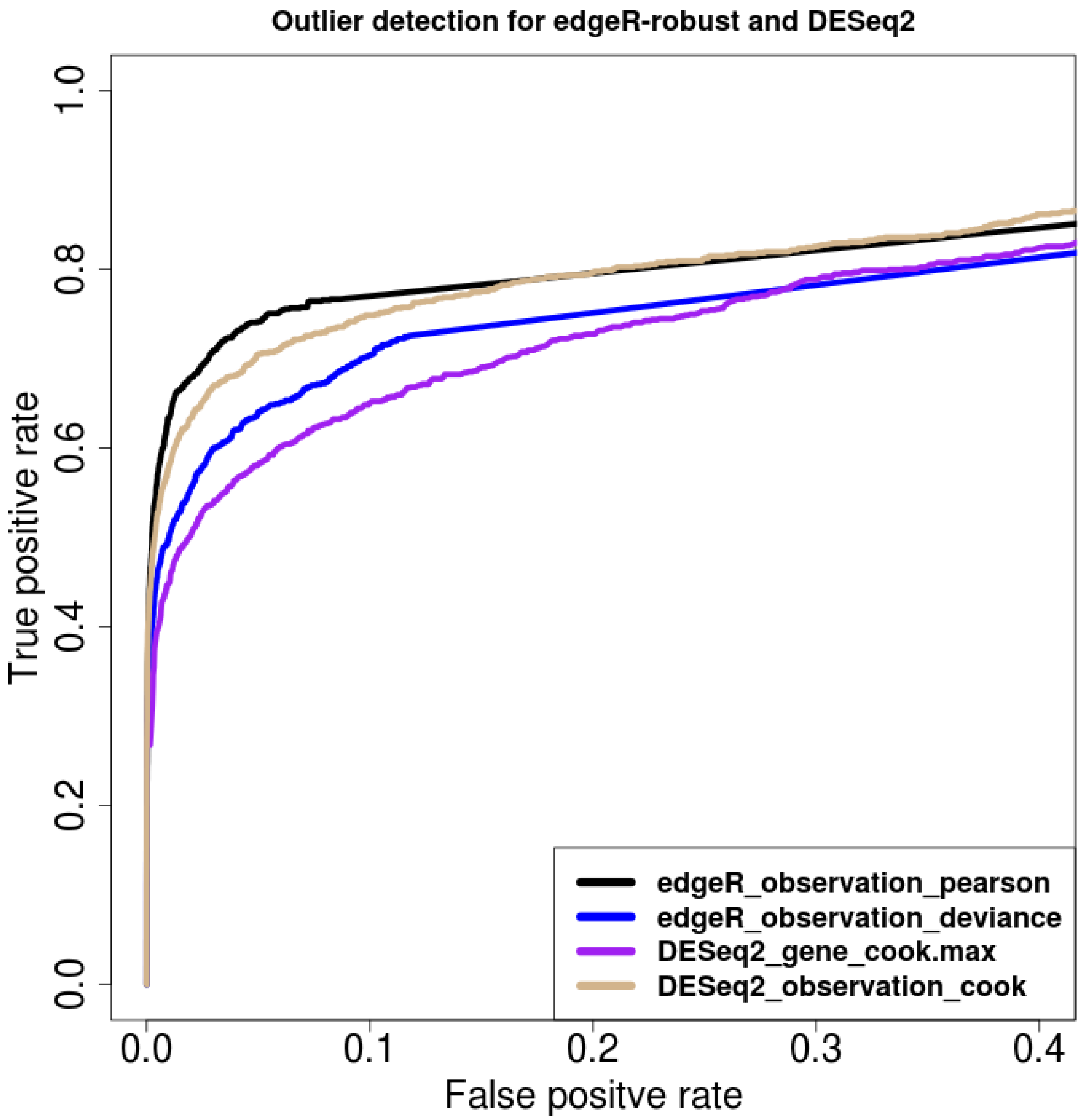

Supplementary Figure 8: Outlier detection of ***edgeR-robust*** and ***DESeq2***. With 10% outliers added by "S" method, we compared how well the observation weights from Pearson residual (black) Deviance residual (blue) in ***edgeR-robust*** and the Cook's distance metric (tan)and maximum of Cook's distance in gene level (purple) in ***DESeq2*** by ROC curve. For gene-level detection, an outlier is detected by gene (i.e. if any observation has an outlier, the gene has an outlier), while at the observation-level, outliers are labeled to the gene and sample.

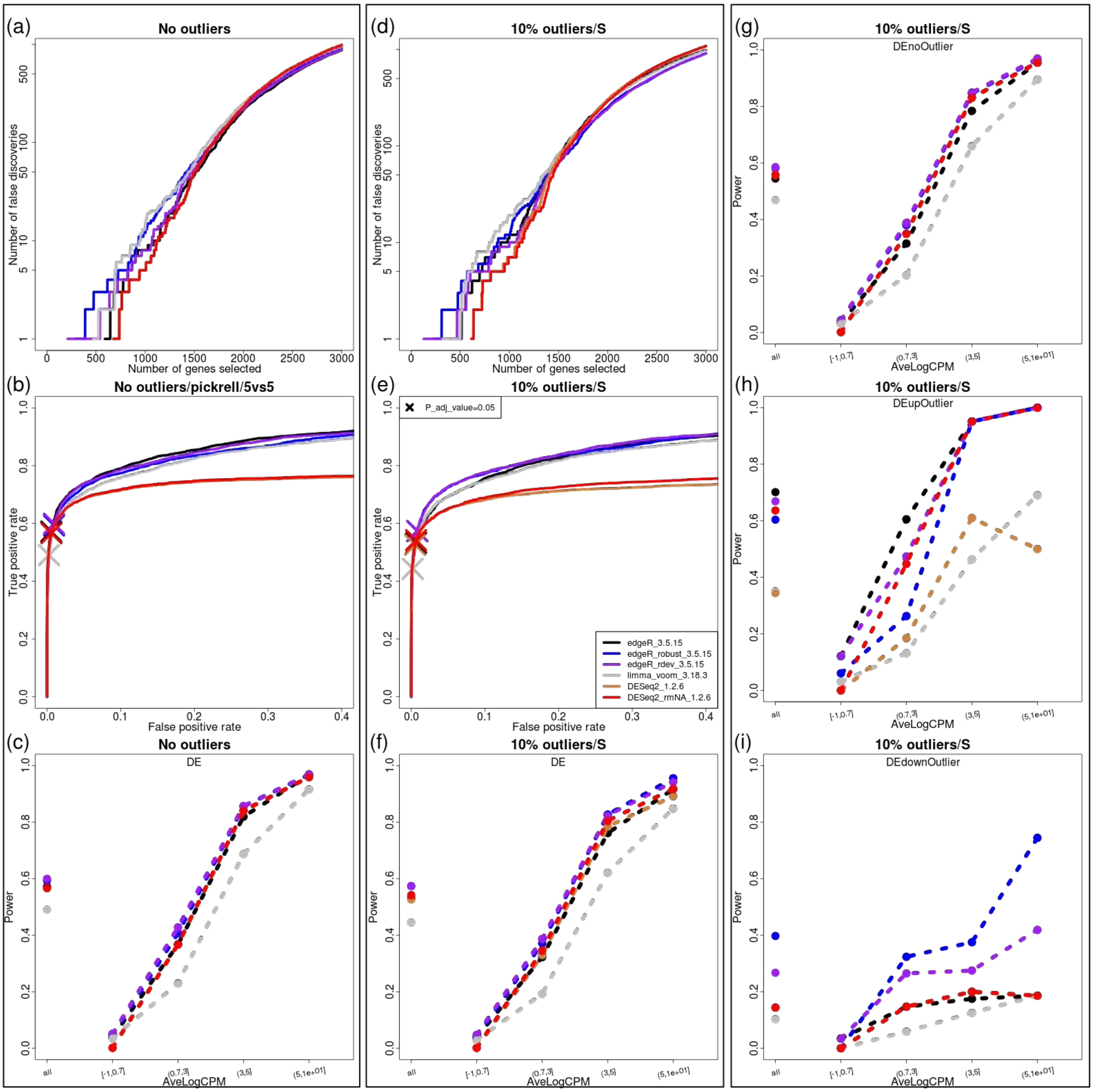

Supplementary Figure 9: Turn off outlier detection in ***DESeq2***. See description from Figure 4 of main paper. For ***DESeq2***, where the outlier detection procedure (Cook's distance) is turned off, denoted ***DESeq-rmNA***. ***edgeR-rdev*** denotes ***edgeR-robust***, where the observation weights are determined using deviance residuals.

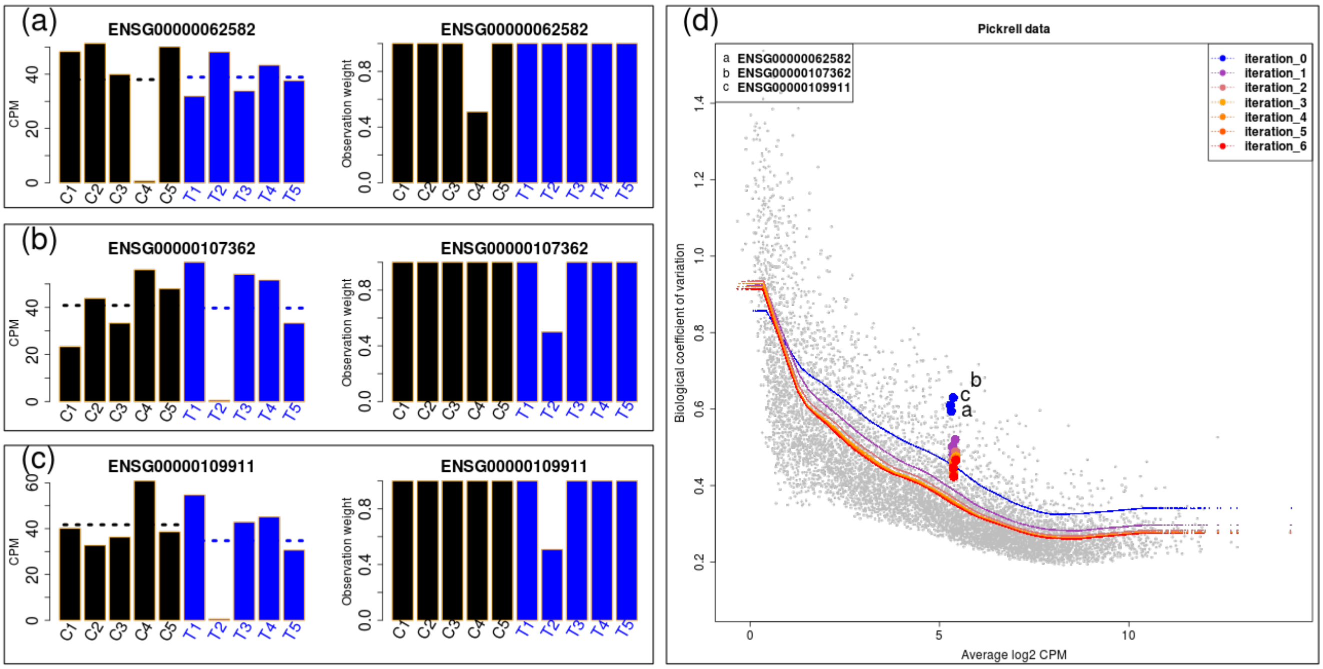

Supplementary Figure 10: Low outlier performance of ***edgeR-robust*** for Pickrell data. (a), (b) and (c) Show the barplots of 3 genes containing an extremely low observation and observation weights claculated using ***edgeR-robust***. (d) The trajectories for all and for the 3 individual genes are shown over 6 iterations of the edgeR-robust reweighted estimation scheme.

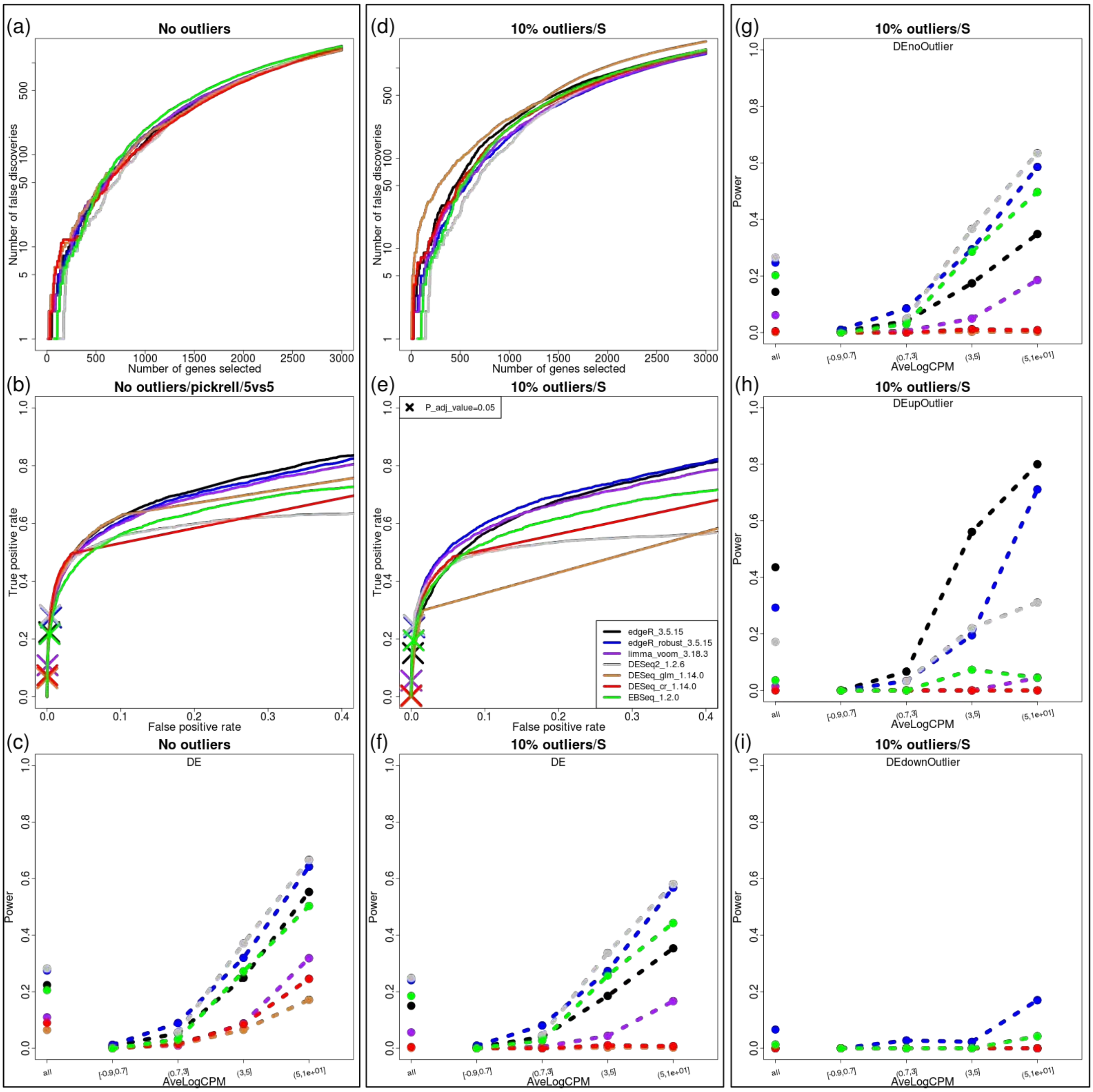

Supplementary Figure 11i: Simulation based on Pickrell dataset: nTags=30000, foldDiff=2, group = 5vs5 , pOutlier=0.1, outlierMech=S, pDiff=0. See description from Figure 4 of main paper.

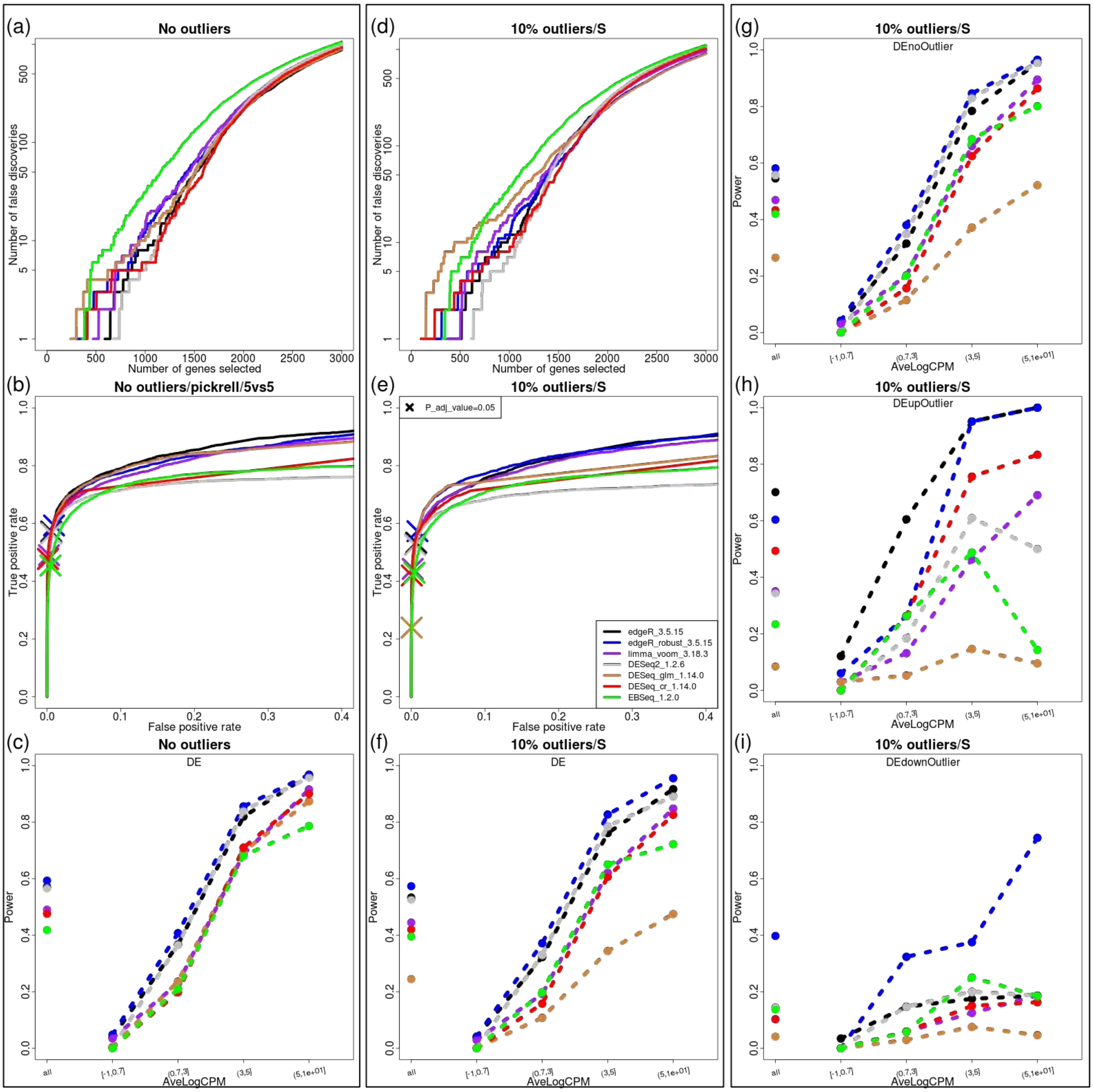

Supplementary Figure 11ii: Simulation based on Pickrell dataset: nTags=30000, foldDiff=3, group = 5vs5 , pOutlier=0.1, outlierMech=S, pDiff=0.1. See description from Figure 4 of main paper.

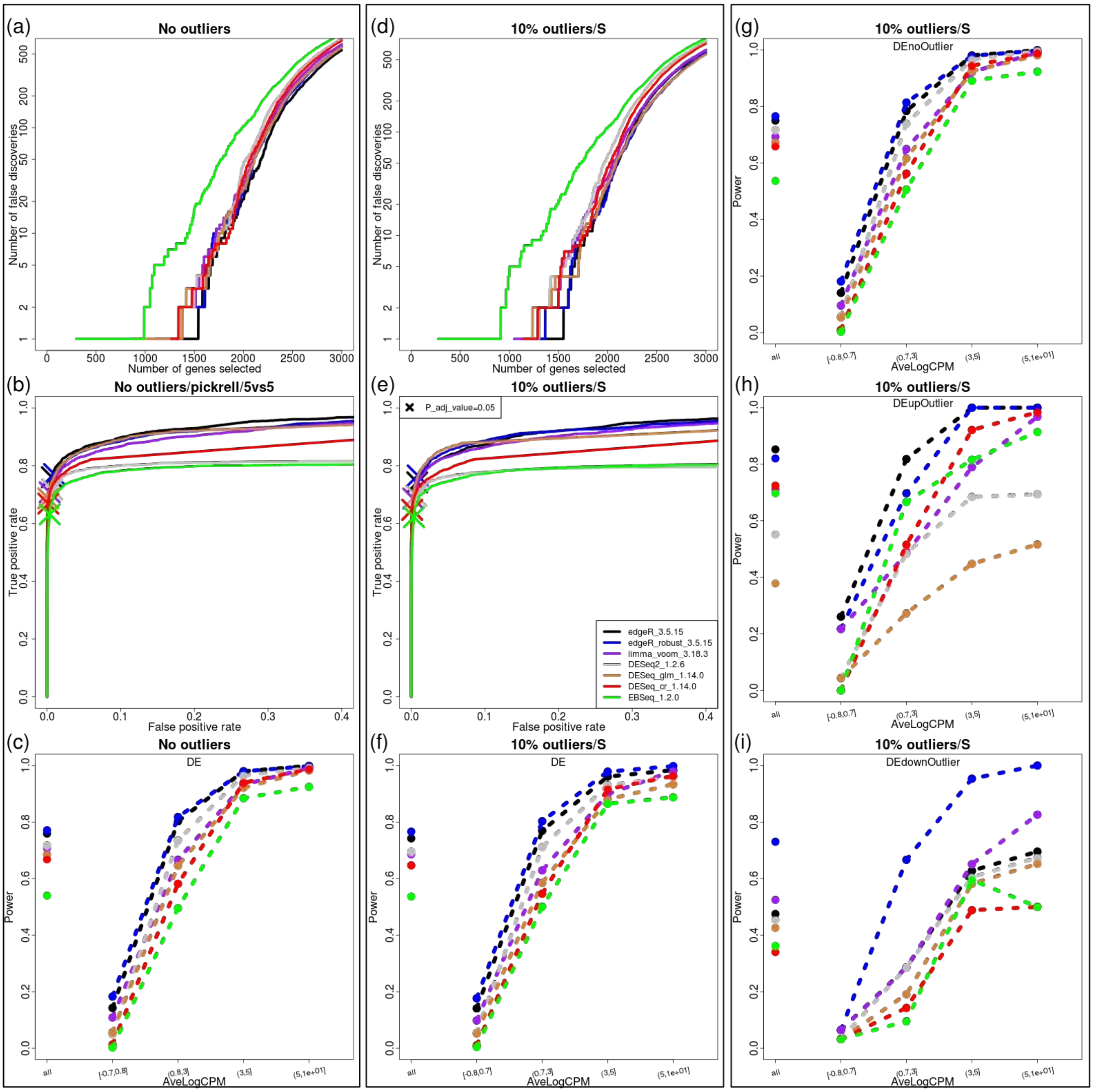

Supplementary Figure 11iii: Simulation based on Pickrell dataset: nTags=30000, foldDiff=6, group = 5vs5 , pOutlier=0.1, outlierMech=S, pDiff=0.1. See description from Figure 4 of main paper.

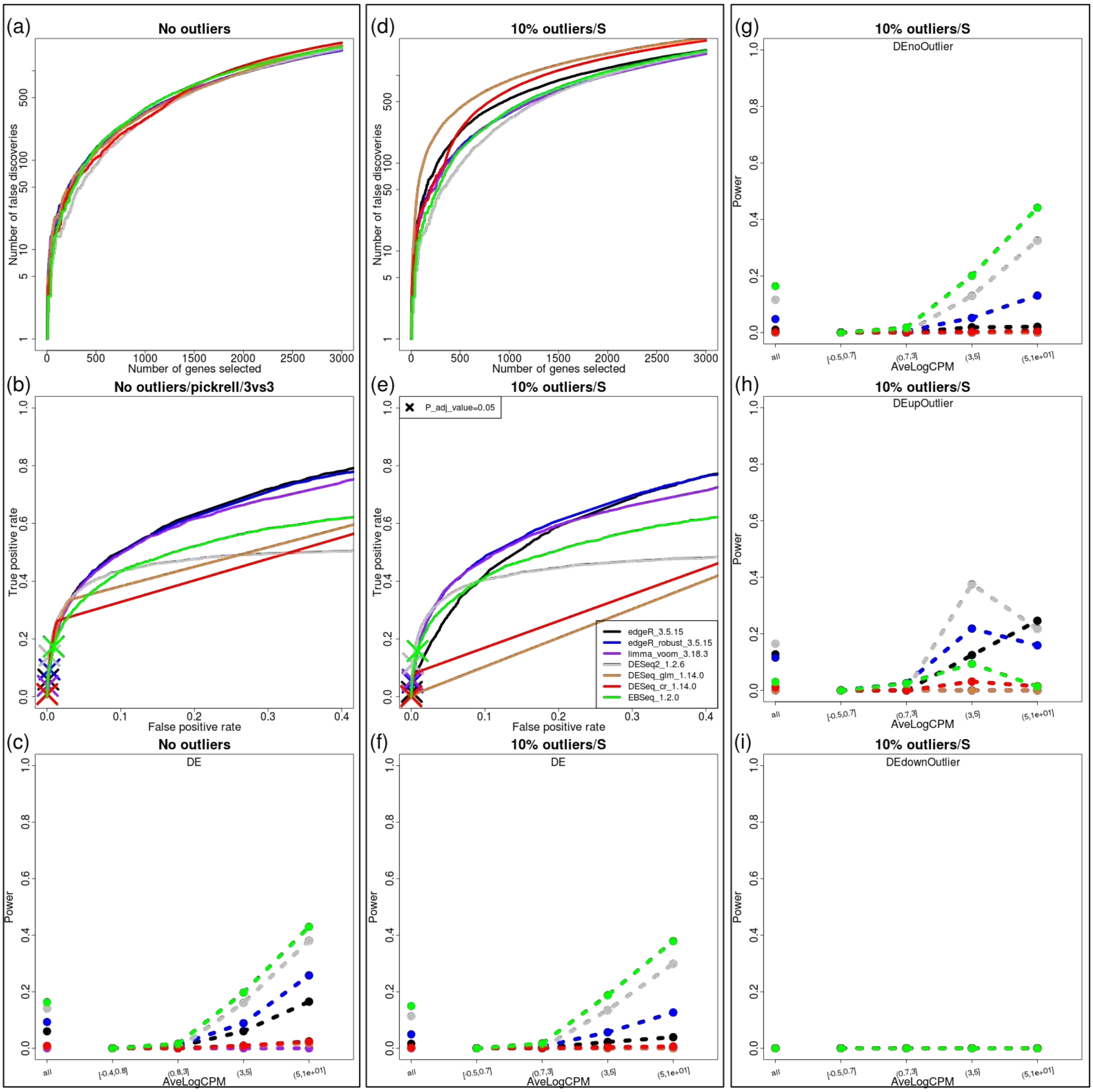

Supplementary Figure 11iv: Simulation based on Pickrell dataset: nTags=30000, foldDiff=2, group = 3vs3 , pOutlier=0.1, outlierMech=S, pDiff=0. See description from Figure 4 of main paper.

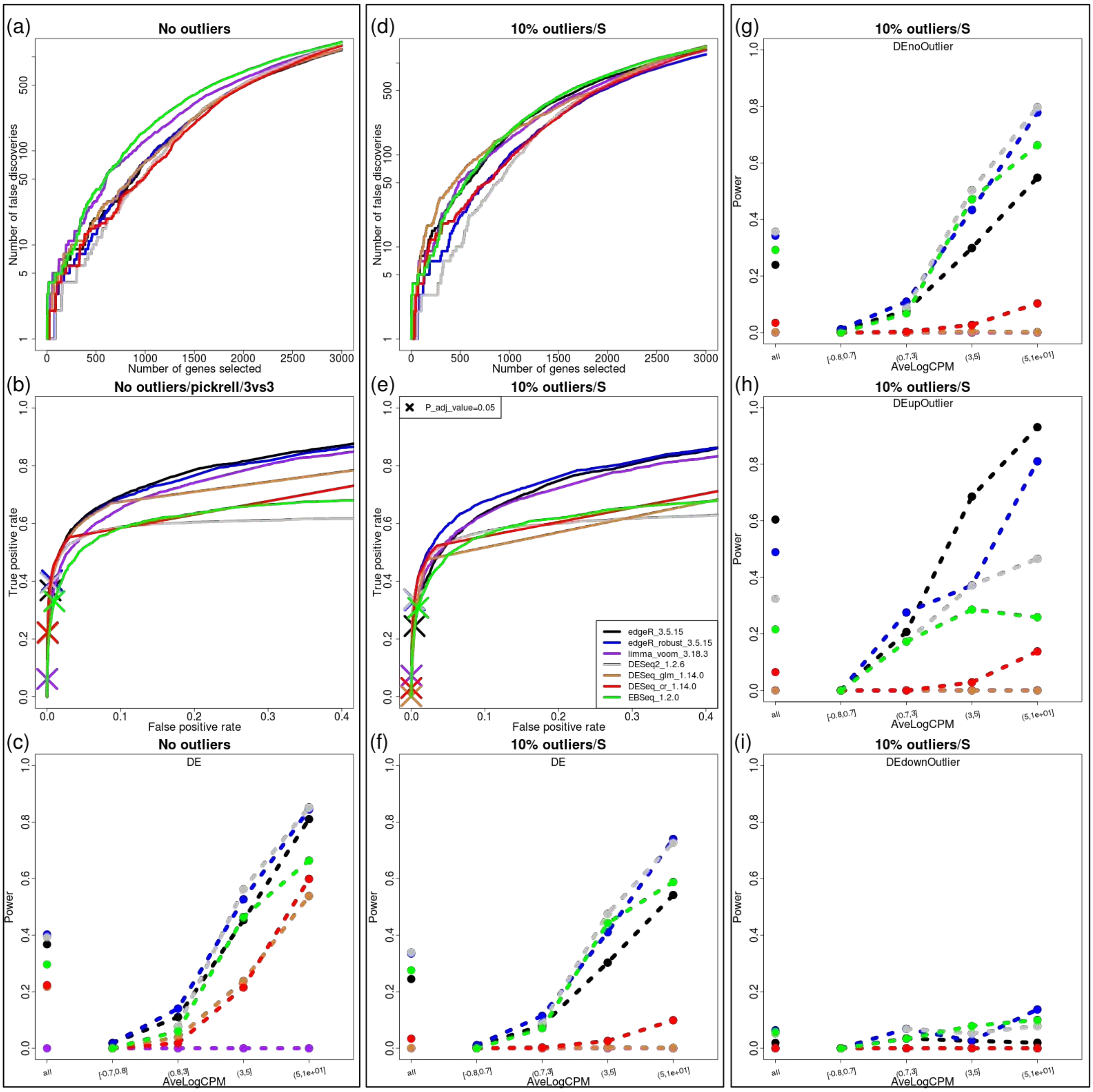

Supplementary Figure 11v: Simulation based on Pickrell dataset: nTags=30000, foldDiff=3, group = 3vs3 , pOutlier=0.1, outlierMech=S, pDiff=0.1. See description from Figure 4 of main paper.

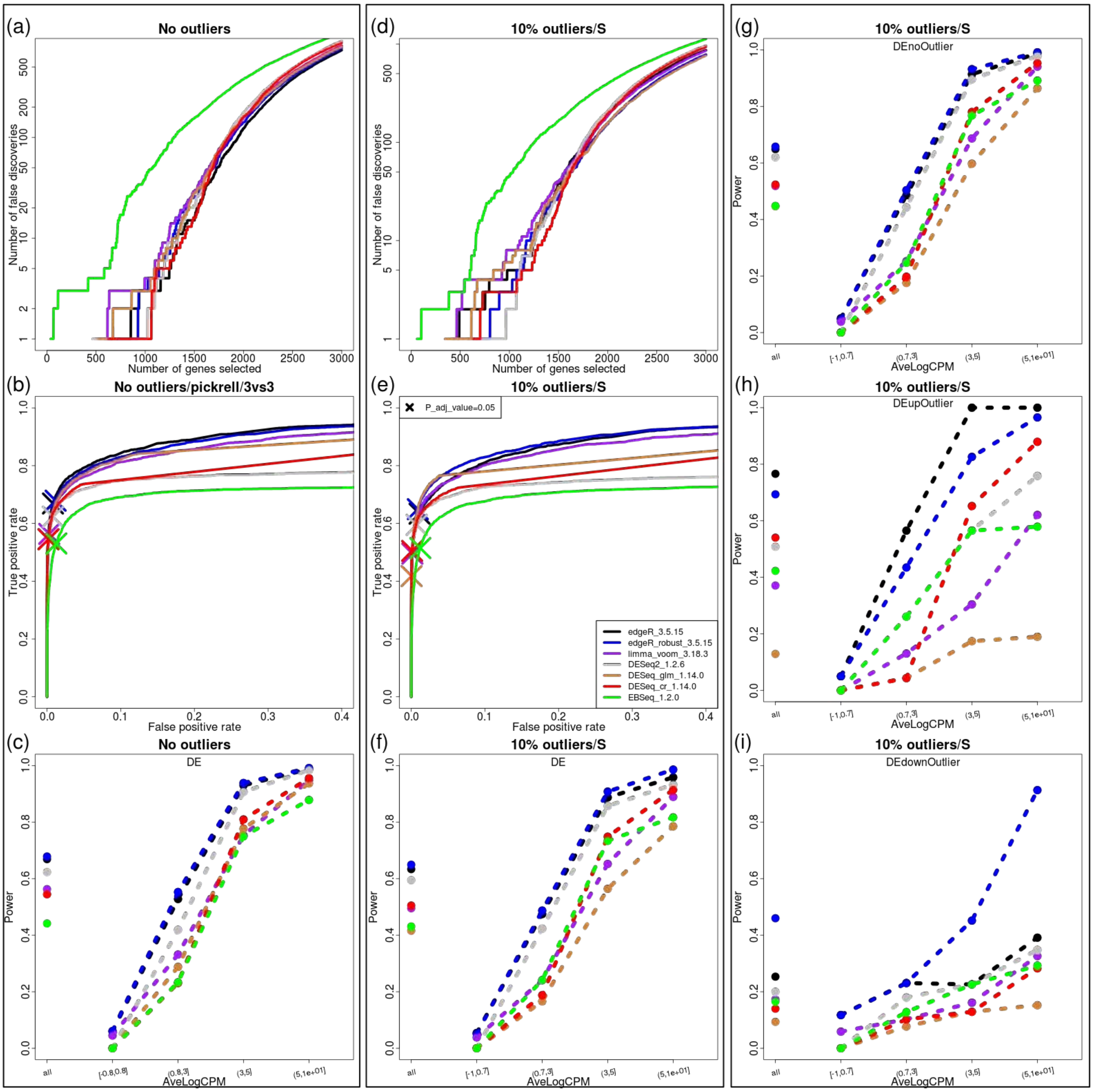

Supplementary Figure 11vi: Simulation based on Pickrell dataset: nTags=30000, foldDiff=6, group = 3vs3 , pOutlier=0.1, outlierMech=S, pDiff=0.1. See description from Figure 4 of main paper.

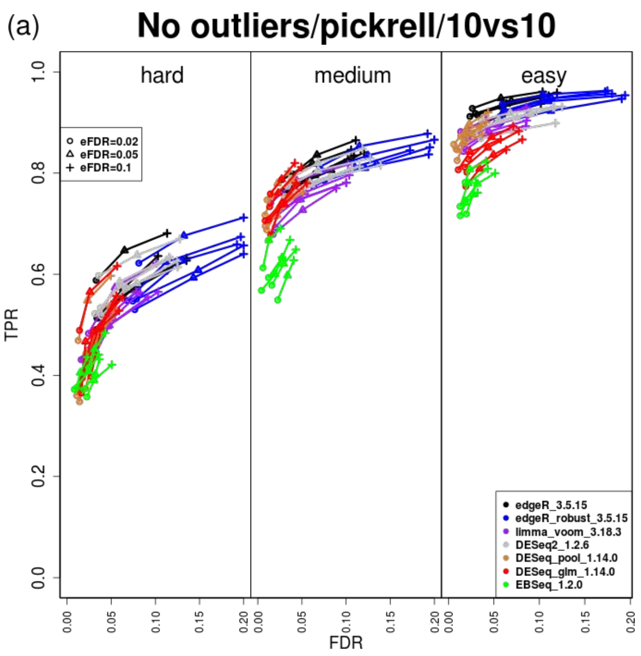

Supplementary Figure 12i: 5 simulations for each setting based on Pickrell dataset: group=10v10, nTags=10000. See description from Figure 5 of main paper.

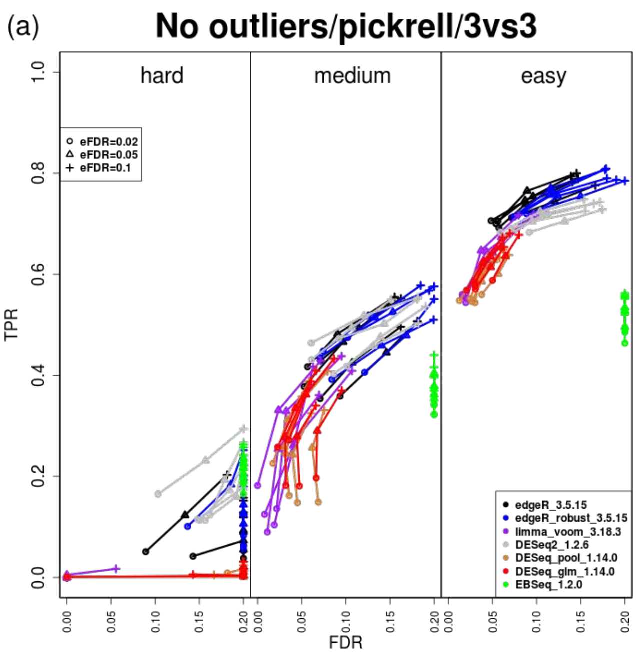

Supplementary Figure 12ii: 5 simulations for each setting based on Pickrell dataset: group=3v3, nTags=10000. See description from Figure 5 of main paper.

2 Rcode

2.1 Rcode for Supplementary Figure

Supplementary Figure 1

```r
n5 <- 10
g5 <- as.factor(rep(0:1, each = n5/2))
data_nofilter <- NBsim(foldDiff = 3,
    dataset = pickrell, group = g5,
    add.outlier = TRUE, pOutlier = 0.1,
    nTags = 10000, drop.extreme.dispersion = FALSE)
max <- quantile(data_nofilter$dataset$dataset.dispersion,
    0.9, names = FALSE)
idx_max <- which(data_nofilter$Dispersion[,
    1] > max)
CPM <- log2((1e+06) * data_nofilter$Lambda[,
    1] + 2)
dispersion <- data_nofilter$Dispersion[,
    1]
dno <- DGEList(counts = data_nofilter$counts,
    group = data_nofilter$group)
colnames(dno) <- paste0(rep(c("C",
    "T"), each = n/2), 1:(n/2))
dno <- calcNormFactors(dno)
dno$genes <- round(cpm(dno, normalized.lib.sizes = TRUE),
    1)
```

```r
# dir is your local directory
# containing Rdata file
load(paste0(dir, "Rdata/supp_manuscript.Rdata"))
library(edgeR)
# sample outlier
par(mar = c(5, 5, 3, 2))
par(oma = c(0, 0, 4, 0))
# barplot
layout(matrix(c(1, 4, 2, 4, 3,
    4), 3, 2, byrow = TRUE), widths = c(0.6,
    1))
tex <- c("(a)", "(b)", "(c)")
k <- 1
i_max <- idx_max[c(1, 50, 100)]
for (j in i_max) {
    col = as.numeric(as.character(dno$sample$group)) *
        3 + 1
    x = barplot(dno$genes[j, ],
        main = rownames(dno)[j],
        col = col, border = "orange",
        cex.main = 2, cex.axis = 2,
        xaxt = "n")
    gm1 <- mean(dno$gene[j, ][dno$sample$group ==
        levels(dno$sample$group)[1]])
    gm2 <- mean(dno$gene[j, ][dno$sample$group ==
```

```
        levels(dno$sample$group)[2]])
    l1 <- sum(dno$sample$group ==
        levels(dno$sample$group)[1])
    l2 <- sum(dno$sample$group ==
        levels(dno$sample$group)[2])
    segments(l1 + 0.9, gm1, 0.1,
        gm1, lty = 3, lwd = 4,
        col = unique(col)[1])
    segments(l1 + l2 + 1.9, gm2,
        l1 + 1.1, gm2, lty = 3,
        lwd = 4, col = unique(col)[2])
    text(cex = 2, x = x - 0.25,
        y = par("usr")[3] - 0.02,
        colnames(dno), pos = 1,
        srt = 60, col = as.numeric(as.character(dno$sample$group)) *
            3 + 1, xpd = TRUE)
    mtext(tex[k], side = 3, adj = -0.13,
        padj = -0.5, cex = 2.5)
    k <- k + 1
}
# edgeR mean dispersion plot
plot(CPM, sqrt(dispersion), pch = 19,
    cex = 0.45, xlab = "Average log2 CPM",
    ylab = "Biological coefficient of variation",
    col = "grey", main = "edgeR",
    cex.main = 3, cex.axis = 2,
    cex.lab = 2, ylim = c(0, 3))
points(CPM[idx_max], sqrt(dispersion[idx_max]),
    pch = 19, cex = 0.45, col = "steelblue")
points(CPM[i_max], sqrt(dispersion[i_max]),
    pch = 19, cex = 2, col = "blue")
points(CPM[i_max] - 0.3, sqrt(dispersion[i_max]) +
    0.02, cex = 2.5, pch = letters[seq(i_max)])
legend("topright", c("90% dispersion",
    "top 10% dispersion", "example genes"),
    col = c("gray", "steelblue",
        "blue"), pch = 19, text.font = 2,
    cex = 1.8)
mtext("(d)", side = 3, adj = -0.13,
    padj = -0.5, cex = 2.5)
resetPar()
```

Supplementary Figure 2

```
source("http://130.60.190.4/robinson_lab/edgeR_robust/robust_simulation.R")
load(paste0(dir, "Rdata/manuscript.Rdata"))
load(paste0(dir, "Rdata/sim.Rdata"))
```

```r
library(pscl)
library(edgeR)
nbfit <- function(counts, zero.infl = TRUE) {
    d <- DGEList(counts = counts)
    d <- calcNormFactors(d)
    d$AveLogCPM <- aveLogCPM(d)
    OffSet <- getOffset(d)
    dispersion <- numeric(nrow(counts))
    zeroComProb <- numeric(nrow(counts))
    idx0 <- c()
    for (i in 1:nrow(counts)) {
        dat <- counts[i, ]
        if (zero.infl) {
            if (any(dat == 0)) {
                f <- zeroinfl(y ~
                  1 | 1, data = data.frame(y = dat),
                  dist = "negbin",
                  offset = OffSet)
                idx0 <- c(idx0,
                  i)
                dispersion[i] <- 1/f$theta
                zeroComProb[i] <- mean(predict(f,
                  type = "zero"))
            } else {
                f <- glm.nb(y ~
                  1 + offset(OffSet),
                  data = data.frame(y = dat))
                dispersion[i] <- 1/f$theta
            }
        } else {
            f <- glm.nb(y ~ 1 +
                offset(OffSet),
                data = data.frame(y = dat))
            dispersion[i] <- 1/f$theta
        }
    }
    output <- list(dispersion = dispersion,
        AveLogCPM = d$AveLogCPM)
    if (zero.infl) {
        zeroProb <- numeric(nrow(counts))
        nbins <- nrow(counts)/20
        group <- cutWithMinN(d$AveLogCPM,
            intervals = nbins,
            min.n = 1)$group
        for (j in 1:nbins) {
            bin <- group == j
            zeroProb[bin] <- 1 -
```

```
                mean(zeroComProb[bin] ==
                  0)
        }
        output$zeroComProb <- zeroComProb
        output$zeroProb <- zeroProb
        output$zeroInd <- idx0
    }
    output
}
cps <- cpm(pickrell)
k <- rowSums(cps >= 1) > 5
pickrell <- pickrell[k, ]
pickrell <- pickrell[1:5000, ]
p_zero <- nbfit(pickrell)
```

```
# dir is your local directory
# containing Rdata file
load(paste0(dir, "Rdata/supp_manuscript.Rdata"))
par(mfrow = c(1, 2))
par(mar = c(5, 5, 1, 2))
par(oma = c(0, 0, 3, 0))
plot(p_zero$AveLogCPM, p_zero$zeroComProb,
    col = "gray", cex = 0.5, xlab = "Average log2 CPM",
    ylab = "Zero component probability",
    cex.axis = 2, cex.lab = 2,
    pch = 19)
points(p_zero$AveLogCPM[p_zero$zeroInd],
    p_zero$zeroComProb[p_zero$zeroInd],
    col = "blue", cex = 0.5, pch = 19)
legend("topright", c("P(zero component) > 0 ",
    "P(zero component) = 0 "),
    col = c("blue", "gray"), pch = 19,
    cex = 2.5)
plot(p_zero$AveLogCPM, sqrt(p_zero$dispersion),
    log = "y", col = "gray", cex = 0.5,
    xlab = "Average log2 CPM",
    ylab = "Biological coefficient of variation",
    ylim = c(0.02, 10), cex.axis = 2,
    cex.lab = 2, pch = 19)
points(p_zero$AveLogCPM[p_zero$zeroInd],
    sqrt(p_zero$dispersion[p_zero$zeroInd]),
    col = "blue", cex = 0.5, pch = 19)
mtext(outer = TRUE, cex = 3, "Zero inflation for Pickrell",
    side = 3, line = 1)
resetPar()
```

Supplementary Figure 3

```
# dir is your local directory
# containing Rdata file
load(paste0(dir, "Rdata/manuscript.Rdata"))
disp <- dw$record$tagwise.dispersion
disp <- do.call("cbind", disp)
dispL <- disp[, -1]
dispF <- disp[, -ncol(disp)]
diff <- abs(dispL - dispF)
ldiff <- log(dispL/dispF)
par(mar = c(7, 5, 3, 2))
par(oma = c(0, 0, 3, 0))
par(mfrow = c(1, 2))
boxplot(diff, ylim = c(0, 0.06),
    cex.main = 1.5, cex.axis = 1.5,
    cex.lab = 1.5, outline = FALSE,
    xaxt = "n", ylab = "absolute change of dispersion")
axis(1, labels = FALSE)
text(1:6, -0.03 * (par("usr")[4] -
    par("usr")[3]) + par("usr")[3],
    srt = 60, adj = 1, labels = colnames(diff),
    xpd = TRUE, cex = 1.5)
boxplot(ldiff, ylim = c(-0.3, 0.1),
    cex.main = 1.5, cex.axis = 1.5,
    cex.lab = 1.5, outline = FALSE,
    xaxt = "n", ylab = "log fold change of dispersion")
axis(1, labels = FALSE)
text(1:6, -0.03 * (par("usr")[4] -
    par("usr")[3]) + par("usr")[3],
    srt = 60, adj = 1, labels = colnames(diff),
    xpd = TRUE, cex = 1.5)
mtext(outer = TRUE, cex = 3, "Convergence of dispersion",
    side = 3, line = 1)
resetPar()
```

Supplementary Figure 4

```
# load(paste0(dir,'Rdata/sim.Rdata'))
ids <- apply(data$S$mask_outlier,
    1, any)
g <- data$group
mm <- data$design
d <- DGEList(data$counts, group = g)
d <- calcNormFactors(d)
d$AveLogCPM <- aveLogCPM(d, prior.count = 1e-05)
d <- estimateGLMCommonDisp(d, design = mm)
d <- estimateGLMTrendedDisp(d,
    design = mm)
```

```
d <- estimateGLMTagwiseDisp(d,
    design = mm)
d1 <- DGEList(data$S$countAddOut,
    group = g)
d1 <- calcNormFactors(d1)
d1$AveLogCPM <- aveLogCPM(d1, prior.count = 1e-05)
d1 <- estimateGLMCommonDisp(d1,
    design = mm)
d1 <- estimateGLMTrendedDisp(d1,
    design = mm)
d1 <- estimateGLMTagwiseDisp(d1,
    design = mm)
```

```
# dir is your local directory
# containing Rdata file
load(paste0(dir, "Rdata/supp_manuscript.Rdata"))
par(mfcol = c(3, 2))
par(mar = c(5, 5, 3, 2))
par(oma = c(0, 0, 4, 0))
plot(data$dataset$dataset.AveLogCPM,
    sqrt(data$dataset$dataset.dispersion),
    pch = 19, cex = 0.45, xlab = "Average log2 CPM",
    ylab = "Biological coefficient of variation",
    col = "grey", main = "No outliers/pickrell/5vs5",
    cex.main = 3, cex.axis = 2,
    cex.lab = 2, ylim = c(0, 1.5))
points(d$AveLogCPM, sqrt(d$tagwise.dispersion),
    col = "steelblue", cex = 0.45,
    pch = 19)
legend("topright", c("original count",
    "simulated count"), col = c("gray",
    "steelblue"), pch = 19, text.font = 2,
    cex = 2.5)
plot(density(data$dataset$dataset.AveLogCPM),
    xlab = "Average log2 CPM",
    ylab = "Density", lwd = 5,
    ylim = c(0, 0.2), main = "",
    cex.axis = 2, cex.lab = 2)
lines(density(d$AveLogCPM), col = "steelblue",
    lwd = 5)
legend("topright", c("original count",
    "simulated count"), col = c("black",
    "steelblue"), lty = 1, text.font = 2,
    cex = 2.5, lwd = 5)
plot(density(sqrt(data$dataset$dataset.dispersion)),
    xlab = "Biological coefficient of variation",
    ylab = "Density", lwd = 5,
```

```
    ylim = c(0, 3), main = "",
    cex.axis = 2, cex.lab = 2)
lines(density(sqrt(d$tagwise.dispersion)),
    col = "steelblue", lwd = 5)
legend("topright", c("original count",
    "simulated count"), col = c("black",
    "steelblue"), lty = 1, text.font = 2,
    cex = 2.5, lwd = 5)
plot(data$dataset$dataset.AveLogCPM,
    sqrt(data$dataset$dataset.dispersion),
    pch = 19, cex = 0.45, xlab = "Average log2 CPM",
    ylab = "Biological coefficient of variation",
    col = "grey", main = "10% outliers/S/pickrell/5vs5",
    cex.main = 3, cex.axis = 2,
    cex.lab = 2, ylim = c(0, 1.5))
points(d1$AveLogCPM[!ids], sqrt(d1$tagwise.dispersion)[!ids],
    col = "steelblue", cex = 0.45,
    pch = 19)
points(d1$AveLogCPM[ids], sqrt(d1$tagwise.dispersion)[ids],
    col = "purple", cex = 0.45,
    pch = 19)
legend("topright", c("original count",
    "simulated count", "outlier"),
    col = c("gray", "steelblue",
        "purple"), pch = 19, text.font = 2,
    cex = 2.5)
plot(density(data$dataset$dataset.AveLogCPM),
    xlab = "Average log2 CPM",
    ylab = "Density", lwd = 5,
    ylim = c(0, 0.2), main = "",
    cex.axis = 2, cex.lab = 2)
lines(density(d1$AveLogCPM[!ids]),
    col = "steelblue", lwd = 5)
lines(density(d1$AveLogCPM[ids]),
    col = "purple", lwd = 5)
legend("topright", c("original count",
    "simulated count", "outlier"),
    col = c("black", "steelblue",
        "purple"), lty = 1, text.font = 2,
    cex = 2.5, lwd = 5)
plot(density(sqrt(data$dataset$dataset.dispersion)),
    xlab = "Biological coefficient of variation",
    ylab = "Density", lwd = 5,
    ylim = c(0, 4), main = "",
    cex.axis = 2, cex.lab = 2)
lines(density(sqrt(d1$tagwise.dispersion[!ids])),
    col = "steelblue", lwd = 5)
```

```
lines(density(sqrt(d1$tagwise.dispersion[ids])),
    col = "purple", lwd = 5)
legend("topright", c("original count",
    "simulated count", "outlier"),
    col = c("black", "steelblue",
        "purple"), lty = 1, text.font = 2,
    cex = 2.5, lwd = 5)
mtext(outer = TRUE, cex = 3, "Validation of simulation model",
    side = 3, line = 1)
resetPar()
```

Supplementary Figure 5

```
library(samr)
library(ggplot2)
library(gridExtra)
library(reshape2)
library(parallel)
library(edgeR)
# Subplot a: Outlier
# distribution in
# Montgomery/Pickrell data
# Model is fit without a
# grouping factor
f1 <- "http://bowtie-bio.sourceforge.net/"
f2 <- "recount/countTables/montpick_count_table.txt"
counts.f <- paste0(f1, f2)
counts.t <- read.table(counts.f,
    header = TRUE, row.names = "gene")
f3 <- "http://bowtie-bio.sourceforge.net"
f4 <- "/recount/phenotypeTables/montpick_phenodata.txt"
pheno.f <- paste0(f3, f4)
pheno <- read.table(pheno.f, header = TRUE)
dge <- DGEList(counts.t)
dge <- dge[(rowSums(cpm(dge) >
    1) >= 10), ]
dge <- calcNormFactors(dge)
dge <- estimateGLMCommonDisp(dge)
dge <- estimateGLMTrendedDisp(dge)
dge <- estimateGLMRobustDisp(dge)
# Format for plotting
m <- melt(dge$weights)
v <- pheno$population
names(v) <- pheno$sample.id
m$Population <- v[m$Var2]
# Shuffle the data so groups
# are more visible
```

```
m <- m[sample(1:nrow(m), nrow(m)),
    ]
ylab <- expression(P(Weight <=
    X))
p1 <- ggplot(m, aes(value, fill = Var2,
    colour = Population)) + stat_ecdf(linetype = "dashed") +
    ylab(ylab) + xlab("Weight") +
    coord_cartesian(ylim = c(0,
        0.5)) + guides(col = guide_legend(ncol = 2,
    override.aes = aes(size = 3))) +
    theme_bw() + theme(legend.key = element_blank(),
    legend.title = element_blank(),
    legend.position = "bottom",
    text = element_text(size = 16),
    axis.line = element_line(size = 0.7,
        color = "black"), plot.margin = unit(c(0,
        0.5, 0, 0.5), "cm"))
p1.label <- textGrob(label = "(a)",
    x = 0.03, y = 0.5, vjust = 0.2,
    gp = gpar(fontsize = 18))
# Subplot b: Compare the
# distribution of weights for
# differentially and
# non-differentially expressed
# genes.  Use SAMseq to decide
# whether a gene is
# differentially expressed for
# subsamples of the Montgomery/
# Pickrell data Select 10
# subsamples containing 10
# individuals from each
# population.
set.seed(42)
getSAMseqSig <- function(sam.result) {
    get.cols <- c("Gene Name",
        "Fold Change", "q-value(%)")
    up.genes <- sam.result$siggenes.table$genes.up
    de.up <- up.genes[which(as.numeric(up.genes[,
        "q-value(%)"]) <= 5), get.cols]
    down.genes <- sam.result$siggenes.table$genes.lo
    de.down <- down.genes[which(as.numeric(down.genes[,
        "q-value(%)"]) <= 5), get.cols]
    result <- rbind(de.up, de.down)
    result
}
t <- counts.t[rowSums(counts.t) >=
    50, ]
```

```
samples <- lapply(c(1:10), function(i,
    p, counts) {
    ceu <- as.vector(sample(p[p$population ==
        "CEU", "sample.id"], 10))
    yri <- as.vector(sample(p[p$population ==
        "YRI", "sample.id"], 10))
    counts[, c(ceu, yri)]
}, pheno, t)
group <- as.factor(rep(c("ceu",
    "yri"), each = 10))
results <- mclapply(samples, function(t,
    group) {
    SAMseq(t, group, resp.type = "Two class unpaired",
        geneid = rownames(t), nperms = 1000,
        nresamp = 1000)
}, group, mc.cores = 16)
sig.results <- mclapply(results,
    getSAMseqSig)
# na.omit because SamSeq
# filters low count genes
wghts <- mapply(function(ids, delist) {
    dge$weights[na.omit(match(delist[,
        "Gene Name"], rownames(dge$weights))),
        colnames(ids)]
}, samples, sig.results)
# Format data for plotting
m <- melt(lapply(wghts, melt))
m$L1 <- as.factor(m$L1)
m <- m[, c("value", "L1")]
colnames(m) <- c("weight", "Replicate")
non.de <- lapply(sig.results, function(sig.res,
    all.res, wghts) {
    wghts[setdiff(all.res, sig.res[,
        "Gene Name"]), ]
}, rownames(dge$weights), dge$weights)
nde <- melt(lapply(non.de, melt))
nde <- nde[, c("value", "L1")]
colnames(nde) <- c("weight", "Replicate")
nde$is.de <- "Not DE"
m$is.de <- "DE"
data <- rbind(nde, m)
# Plot
p2 <- ggplot(data, aes(weight,
    fill = Replicate, colour = is.de)) +
    stat_ecdf() + ylab(ylab) +
    xlab("Weight") + coord_cartesian(ylim = c(0,
    0.5)) + theme_bw() + theme(legend.title = element_blank(),
```

```
    legend.position = "bottom",
    legend.key = element_blank(),
    text = element_text(size = 16),
    axis.line = element_line(size = 0.7,
        color = "black"), plot.margin = unit(c(0,
        0.5, 0, 0.5), "cm"))
p2.label <- textGrob(label = "(b)",
    x = 0.03, y = 0.5, vjust = 0.2,
    gp = gpar(fontsize = 18))
```

```
library(ggplot2)
library(gridExtra)
# dir is your local directory
# containing Rdata file
load(paste0(dir, "Rdata/supp_weights.Rdata"))
grid.arrange(p1.label, p2.label,
    p1, p2, ncol = 2, heights = c(2,
        25), clip = FALSE)
```

Supplementary Figure 6

```
# dir is your local directory
# containing Rdata file
load(paste0(dir, "Rdata/manuscript.Rdata"))
idx <- apply(dw$weights, 1, function(x) sum(x <=
    0.5) == 1)
ids <- which(apply(dw$weights,
    1, function(x) sum(x <= 0.5) ==
        1))
idx0.5 <- dw$weights < 0.5
idx0.5[!idx, ] <- FALSE
d_out <- d_non <- dw
d_out$weights <- d_non$weights <- matrix(1,
    nrow(dw$counts), ncol(dw$counts))
d_non$weights[idx0.5] <- 1e-06
d_non$CPM <- rowSums(d_non$genes *
    d_non$weights)/rowSums(d_non$weights)
d_out$weights[!idx0.5] <- 1e-06
d_out$CPM <- rowSums(d_out$genes *
    d_out$weights)/rowSums(d_out$weights)
ratio <- log2(d_out$CPM[ids]/d_non$CPM[ids])
dr <- density(ratio)
ids1 <- which(rank(abs(ratio -
    0)) <= 3)
dw$weights[ids, ][ids1, ]
dw$genes[ids, ][ids1, ]
```

```
aDensity <- function(density, x.min,
    y.min) {
    library(zoo)
    dr <- density
    xt.min <- diff(dr$x[dr$x <=
        x.min])
    yt.min <- rollmean(dr$y[dr$x <=
        x.min], 2)
    xt.max <- diff(dr$x[dr$x <=
        x.max])
    yt.max <- rollmean(dr$y[dr$x <=
        x.max], 2)
    sum(xt.max * yt.max) - sum(xt.min *
        yt.min)
}
par(mar = c(5, 5, 3, 2))
par(oma = c(0, 0, 4, 0))
x.min <- log2(1.5)
x.max <- log2(10)
ids_cut <- cut(d$AveLogCPM[ids],
    4)
par(mfrow = c(2, 2))
for (i in levels(ids_cut)) {
    ratio_cut <- ratio[ids_cut ==
        i]
    dr_cut <- density(ratio_cut)
    area_cut <- aDensity(dr_cut,
        x.min, x.max)
    plot(dr_cut, xlim = c(-1, 5),
        lwd = 4, xlab = "r", cex.main = 2,
        cex.axis = 2, cex.lab = 2,
        main = paste0("AveLogCPM=",
            i), ylim = c(0, 1.2))
    polygon(c(x.min, dr_cut$x[dr_cut$x >
        x.min & dr_cut$x < x.max],
        x.max), c(0, dr_cut$y[dr_cut$x >=
        x.min & dr_cut$x <= x.max],
        0), col = "blue")
    legend("topright", paste0("log2(1.5)<r<log2(10)=",
        round(area_cut, 2)), col = "blue",
        pch = 19, text.font = 2,
        cex = 2)
    abline(h = 0, lwd = 6, col = "gray")
}
mtext(outer = TRUE, cex = 2, "log2 of the ratio of outlier to non-outlier CPM",
    side = 3, line = 1)
resetPar()
```

Supplementary Figure 7

```
# load(paste0(dir,'Rdata/sim.Rdata'))
library(DESeq)
grp <- data$group
counts <- data$counts
de <- newCountDataSet(counts, grp)
de <- estimateSizeFactors(de)
de <- estimateDispersions(de, method = "pooled")
res <- nbinomTest(de, levels(grp)[1],
    levels(grp)[2])
de0 <- newCountDataSet(data$S$countAddOut,
    grp)
de0 <- estimateSizeFactors(de0)
de0 <- estimateDispersions(de0,
    method = "pooled")
res0 <- nbinomTest(de0, levels(grp)[1],
    levels(grp)[2])
dsp <- featureData(de)@data$disp_pooled
dsp0 <- featureData(de0)@data$disp_pooled
o <- rowSums(data$S$mask_outlier) >
    0
de_cr <- estimateDispersions(de,
    method = "pooled-CR")
res_cr <- nbinomTest(de_cr, levels(grp)[1],
    levels(grp)[2])
de0_cr <- estimateDispersions(de0,
    method = "pooled-CR")
res0_cr <- nbinomTest(de0_cr, levels(grp)[1],
    levels(grp)[2])
dsp_cr <- featureData(de_cr)@data$disp_pooled
dsp0_cr <- featureData(de0_cr)@data$disp_pooled
```

```
# dir is your local directory
# containing Rdata file
load(paste0(dir, "Rdata/supp_manuscript.Rdata"))
par(mfcol = c(2, 2))
par(mar = c(5, 5, 3, 2))
par(oma = c(0, 0, 4, 0))
plot(res$baseMean, dsp, log = "xy",
    xlab = "log(baseMean)", ylab = "log(Dispersion)",
    pch = 19, cex = 0.45, col = "grey",
    cex.main = 3, cex.axis = 2,
    cex.lab = 2, ylim = c(0.1,
        20), main = "DESeq (pooled)")
points(res0$baseMean, dsp0, col = "blue",
    pch = 19, cex = 0.45)
```

```
legend("topright", c("no outliers",
    "with outliers"), col = c("gray",
    "blue"), pch = 19, text.font = 2,
    cex = 2.5)
plot(dsp, dsp0, log = "xy", col = c("gray",
    "blue")[o + 1], xlab = "log(Dispersion) (no outliers)",
    ylab = "log(Dispersion) (with outliers)",
    pch = 19, cex = 0.45, cex.main = 3,
    cex.axis = 2, cex.lab = 2,
    ylim = c(0.2, 10), main = "DESeq (pooled)")
legend("bottomright", c("no outliers",
    "outliers"), col = c("gray",
    "blue"), pch = 19, text.font = 2,
    cex = 2.5)
plot(res_cr$baseMean, dsp_cr, log = "xy",
    xlab = "log(baseMean)", ylab = "log(Dispersion)",
    pch = 19, cex = 0.45, col = "grey",
    cex.main = 3, cex.axis = 2,
    cex.lab = 2, ylim = c(0.1,
        20), main = "DESeq (pooled-CR)")
points(res0_cr$baseMean, dsp0_cr,
    col = "blue", pch = 19, cex = 0.45)
legend("topright", c("no outliers",
    "with outliers"), col = c("gray",
    "blue"), pch = 19, text.font = 2,
    cex = 2.5)
plot(dsp_cr, dsp0_cr, log = "xy",
    col = c("gray", "blue")[o +
        1], xlab = "log(Dispersion) (no outliers)",
    ylab = "log(Dispersion) (with outliers)",
    pch = 19, cex = 0.45, cex.main = 3,
    cex.axis = 2, cex.lab = 2,
    ylim = c(0.2, 10), main = "DESeq (pooled-CR)")
legend("bottomright", c("no outliers",
    "outliers"), col = c("gray",
    "blue"), pch = 19, text.font = 2,
    cex = 2.5)
resetPar()
```

Supplementary Figure 8

```
# load(paste0(dir,'Rdata/sim.Rdata'))
library(DESeq2)
library(edgeR)
findOutlier <- function(y, outlierMech = c("S",
    "R", "M")) {
    outlierMech <- match.arg(outlierMech,
```

```
        c("S", "R", "M"))
    counts <- y[[outlierMech]][["countAddOut"]]
    group <- y[["group"]]
    design <- y[["design"]]
    mask_outlier <- y[[outlierMech]][["mask_outlier"]]
    mask_outlier_gene <- apply(y[[outlierMech]][["mask_outlier"]],
        1, any)
    DESeq2 <- {
        library(DESeq2)
        colData <- data.frame(group)
        dse <- DESeqDataSetFromMatrix(countData = counts,
            colData = colData,
            design = ~group)
        colData(dse)$group <- as.factor(colData(dse)$group)
        dse <- DESeq(dse)
        res <- results(dse)
        pred_mask_outlier <- is.na(res$pvalue)
        cook <- assays(dse)[["cooks"]]
        cook.max <- mcols(dse)$maxCooks
        m <- nrow(attr(dse, "modelMatrix"))
        p <- ncol(attr(dse, "modelMatrix"))
        cutoff <- seq(0.001, 0.9999,
            by = 0.001)
        cutoff <- sapply(cutoff,
            function(x) qf(x, p,
                m - p))
        list(cook = cook, cook.max = cook.max,
            cutoff = cutoff, pred_mask_outlier = pred_mask_outlier)
    }
    edgeR <- {
        library(edgeR)
        d <- DGEList(counts = counts,
            group = group)
        d <- calcNormFactors(d)
        dw <- estimateGLMRobustDisp(d,
            design = design, prior.df = 10,
            maxit = 6)
        dwd <- estimateGLMRobustDisp(d,
            design = design, prior.df = 10,
            maxit = 6, residual.type = "deviance")
        list(pea = dw$weights,
            dev = dwd$weights)
    }
    list(mask_outlier = mask_outlier,
        mask_outlier_gene = mask_outlier_gene,
        DESeq2 = DESeq2, edgeR = edgeR)
}
```

```
outTable <- function(mask_pred,
    mask_outlier) {
    pred <- as.factor(rep("n",
        length(mask_pred)))
    levels(pred) <- c("n", "p")
    pred[mask_pred] <- "p"
    label <- as.factor(rep("N",
        length(mask_outlier)))
    levels(label) <- c("N", "P")
    label[mask_outlier] <- "P"
    tab <- table(pred, label)
    TP <- tab["p", "P"]
    TN <- tab["n", "N"]
    FP <- tab["p", "N"]
    FN <- tab["n", "P"]
    tpr <- TP/(TP + FN)
    fpr <- FP/(FP + TN)
    output <- list(table = tab,
        tpr = tpr, fpr = fpr)
}
findROC_DESeq2_gene <- function(y,
    mask_outlier, cutoff) {
    tpr <- fpr <- tab <- list()
    for (i in cutoff) {
        mask_i <- y > i
        mask_i[is.na(mask_i)] <- TRUE
        T <- outTable(mask_i, mask_outlier)
        tpr <- c(tpr, T$tpr)
        fpr <- c(fpr, T$fpr)
    }
    tpr <- unlist(tpr)
    fpr <- unlist(fpr)
    list(tpr = tpr, fpr = fpr)
}
findROC_matrix <- function(y, mask_outlier,
    inverse = FALSE) {
    library(ROCR)
    y <- as.vector(y)
    if (inverse)
        y <- 1 - y
    mask <- which(as.vector(mask_outlier))
    label <- as.factor(rep("nonDE",
        length(y)))
    levels(label) <- c("nonDE",
        "DE")
    label[mask] <- "DE"
    pred <- prediction(y, label,
```

```
        label.ordering = c("nonDE",
            "DE"))
    perf <- performance(pred, "tpr",
        "fpr")
    list(tpr = perf@y.values[[1]],
        fpr = perf@x.values[[1]])
}
out <- findOutlier(data)  #data comes from sim.Rdata
out_DESeq2_gene <- findROC_DESeq2_gene(out$DESeq2$cook.max,
    out$mask_outlier_gene, out$DESeq2$cutoff)
out_DESeq2_matrix <- findROC_matrix(out$DESeq2$cook,
    out$mask_outlier)
out_edgeR_matrix_pea <- findROC_matrix(out$edgeR$pea,
    out$mask_outlier, inverse = TRUE)
out_edgeR_matrix_dev <- findROC_matrix(out$edgeR$dev,
    out$mask_outlier, inverse = TRUE)
```

```
# dir is your local directory
# containing Rdata file
load(paste0(dir, "Rdata/supp_manuscript.Rdata"))
par(oma = c(0, 1, 0, 0))
par(mar = c(5, 5, 3, 2))
plot(out_edgeR_matrix_pea$fpr,
    out_edgeR_matrix_pea$tpr, ylab = "True positive rate",
    xlab = "False positve rate",
    lwd = 5, type = "l", cex.main = 1.5,
    cex.axis = 2, cex.lab = 2,
    xlim = c(0, 0.4), main = "Outlier detection for edgeR-robust and DESeq2")
lines(out_edgeR_matrix_dev$fpr,
    out_edgeR_matrix_dev$tpr, col = "blue",
    lwd = 5)
lines(out_DESeq2_gene$fpr, out_DESeq2_gene$tpr,
    col = "purple", lwd = 5)
lines(out_DESeq2_matrix$fpr, out_DESeq2_matrix$tpr,
    col = "tan", lwd = 5)
legend("bottomright", c("edgeR_observation_pearson",
    "edgeR_observation_deviance",
    "DESeq2_gene_cook.max", "DESeq2_observation_cook"),
    col = c("black", "blue", "purple",
        "tan"), lty = 1, lwd = 7,
    text.font = 2, cex = 1.5)
resetPar()
```

Supplementary Figure 9

```
# dir is your local directory
# containing Rdata file
load(paste0(dir, "Rdata/supp_sim.Rdata"))
s.method <- c("edgeR", "edgeR_robust",
    "edgeR_rdev", "limma_voom",
    "DESeq2", "DESeq2_rmNA")
summPlot(pval_b_p5v5lf3, pval_s_p5v5lf3,
    byAveLogCPM = TRUE, selected.method = s.method)
```

Supplementary Figure 10

```
n <- 10
g <- as.factor(rep(0:1, each = n/2))
mm <- model.matrix(~g)
set.seed(100)
s <- sample(ncol(pickrell), n)
cnt <- pickrell[, s]
colnames(cnt) <- paste0(rep(c("C",
    "T"), each = n/2), 1:(n/2))
ids <- c("ENSG00000062582", "ENSG00000107362",
    "ENSG00000109911")
cntl <- cnt
cntl[ids, ][1, 4] <- 1
cntl[ids, ][2, 7] <- 1
cntl[ids, ][3, 7] <- 1
dl <- DGEList(counts = cntl, group = g)
dl <- calcNormFactors(dl)
cpl <- round(cpm(dl, normalized.lib.sizes = TRUE),
    1)
dl$genes <- cpl
dl <- dl[rowSums(cpl > 1) >= 2,
    ]
dl <- estimateGLMCommonDisp(dl,
    design = mm)
dl <- estimateGLMTrendedDisp(dl,
    design = mm, method = "bin.loess")
dl <- estimateGLMTagwiseDisp(dl,
    design = mm)
dlw <- estimateGLMRobustDisp(dl,
    design = mm, maxit = 6, record = TRUE)
```

```
# dir is your local directory
# containing Rdata file
load(paste0(dir, "Rdata/supp_manuscript.Rdata"))
par(mar = c(5, 5, 3, 2))
par(oma = c(0, 0, 1, 0))
```

```
id <- c("ENSG00000062582", "ENSG00000107362",
    "ENSG00000109911")
idx <- match(id, rownames(dlw))
# make trend color
Lab.palette <- colorRampPalette(c("blue",
    "orange", "red"), space = "Lab")
# barplot
layout(matrix(c(1, 4, 7, 2, 5,
    7, 3, 6, 7), 3, 3, byrow = TRUE),
    widths = c(0.5, 0.5, 1))
tex <- c("(a)", "(b)", "(c)")
k <- 1
for (j in idx) {
    col = as.numeric(as.character(dlw$sample$group)) *
        3 + 1
    x = barplot(dlw$genes[j, ],
        main = rownames(dlw)[j],
        col = col, border = "orange",
        cex.main = 2, cex.axis = 2,
        xaxt = "n", ylab = "CPM",
        cex.lab = 1.5)
    gm1 <- mean(dlw$gene[j, ][dlw$sample$group ==
        levels(dlw$sample$group)[1]])
    gm2 <- mean(dlw$gene[j, ][dlw$sample$group ==
        levels(dlw$sample$group)[2]])
    l1 <- sum(dlw$sample$group ==
        levels(dlw$sample$group)[1])
    l2 <- sum(dlw$sample$group ==
        levels(dlw$sample$group)[2])
    segments(l1 + 0.9, gm1, 0.1,
        gm1, lty = 3, lwd = 4,
        col = unique(col)[1])
    segments(l1 + l2 + 1.9, gm2,
        l1 + 1.1, gm2, lty = 3,
        lwd = 4, col = unique(col)[2])
    text(cex = 2, x = x - 0.25,
        y = par("usr")[3] - 1,
        colnames(dlw), pos = 1,
        srt = 60, col = as.numeric(as.character(dlw$sample$group)) *
            3 + 1, xpd = TRUE)
    mtext(tex[k], side = 3, adj = -0.13,
        padj = -0.5, cex = 2.5)
    k <- k + 1
}
k <- 1
for (j in idx) {
    x <- barplot(dlw$weights[j,
```

```
        ], main = rownames(dlw)[j],
        col = col, border = "orange",
        cex.main = 2, cex.axis = 2,
        xaxt = "n", ylab = "Observation weight",
        cex.lab = 1.5)
    text(cex = 2, x = x - 0.25,
        y = par("usr")[3], colnames(dlw),
        pos = 1, srt = 60, col = as.numeric(as.character(dlw$sample$group)) *
            3 + 1, xpd = TRUE)
    k <- k + 1
}
Lab.palette <- colorRampPalette(c("blue",
    "orange", "red"), space = "Lab")
plot(dlw$AveLogCPM, sqrt(dlw$tagwise.dispersion),
    pch = 19, cex = 0.45, xlab = "Average log2 CPM",
    ylab = "Biological coefficient of variation",
    col = "grey", main = "Pickrell data",
    cex.main = 1.5, cex.axis = 1.5,
    cex.lab = 1.5, ylim = c(0.1,
        1.5))
mapply(function(u, v, w) points(u,
    sqrt(v), col = w, pch = 16,
    cex = 0.45), u = dlw$record$AveLogCPM,
    v = dlw$record$trended.dispersion,
    w = Lab.palette(length(dlw$record$AveLogCPM)))
mapply(function(u, v, w) points(u[idx],
    sqrt(v[idx]), col = w, pch = 16,
    cex = 2.5), u = dlw$record$AveLogCPM,
    v = dlw$record$tagwise.dispersion,
    w = Lab.palette(length(dlw$record$AveLogCPM)))
points(dlw$record$AveLogCPM[[1]][idx] +
    c(0.4, 0.6, 0.4), sqrt(dlw$record$tagwise.dispersion[[1]][idx]) +
    c(0, 0.05, 0.025), cex = 2.5,
    pch = letters[seq(idx)])
legend("topright", names(dlw$record$trended.dispersion),
    col = Lab.palette(length(dlw$record$AveLogCPM)),
    pch = 19, lty = 3, cex = 1.5,
    text.font = 2)
legend("topleft", id, col = 1,
    pch = letters[seq(id)], cex = 1.5,
    text.font = 2)
mtext("(d)", side = 3, adj = -0.06,
    padj = -0.5, cex = 2.5)
par(xpd = NA)
rect(grconvertX(0.0025, from = "ndc"),
    grconvertY(0.695, from = "ndc"),
    grconvertX(0.49, from = "ndc"),
```

```
    grconvertY(0.998, from = "ndc"),
    lwd = 1.5)
rect(grconvertX(0.0025, from = "ndc"),
    grconvertY(0.36, from = "ndc"),
    grconvertX(0.49, from = "ndc"),
    grconvertY(0.67, from = "ndc"),
    lwd = 1.5)
rect(grconvertX(0.0025, from = "ndc"),
    grconvertY(0.01, from = "ndc"),
    grconvertX(0.49, from = "ndc"),
    grconvertY(0.338, from = "ndc"),
    lwd = 1.5)
rect(grconvertX(0.5, from = "ndc"),
    grconvertY(0.01, from = "ndc"),
    grconvertX(0.99, from = "ndc"),
    grconvertY(0.998, from = "ndc"),
    lwd = 1.5)
resetPar()
```

Supplementary Figure 11i

```
# dir is your local directory
# containing Rdata file
load(paste0(dir, "Rdata/supp_sim.Rdata"))
s.method <- c("edgeR", "edgeR_robust",
    "limma_voom", "DESeq2", "DESeq_glm",
    "DESeq_cr", "EBSeq")
summPlot(pval_b_p5v5lf2, pval_s_p5v5lf2,
    byAveLogCPM = TRUE, selected.method = s.method)
```

Supplementary Figure 11ii

```
# dir is your local directory
# containing Rdata file
# load(paste0(dir,
# 'Rdata/supp_sim.Rdata'))
s.method <- c("edgeR", "edgeR_robust",
    "limma_voom", "DESeq2", "DESeq_glm",
    "DESeq_cr", "EBSeq")
summPlot(pval_b_p5v5lf3, pval_s_p5v5lf3,
    byAveLogCPM = TRUE, selected.method = s.method)
```

Supplementary Figure 11iii

```
# dir is your local directory
# containing Rdata file
# load(paste0(dir,
# 'Rdata/supp_sim.Rdata'))
s.method <- c("edgeR", "edgeR_robust",
    "limma_voom", "DESeq2", "DESeq_glm",
    "DESeq_cr", "EBSeq")
summPlot(pval_b_p5v5lf6, pval_s_p5v5lf6,
    byAveLogCPM = TRUE, selected.method = s.method)
```

Supplementary Figure 11iv

```
# dir is your local directory
# containing Rdata file
# load(paste0(dir,
# 'Rdata/supp_sim.Rdata'))
s.method <- c("edgeR", "edgeR_robust",
    "limma_voom", "DESeq2", "DESeq_glm",
    "DESeq_cr", "EBSeq")
summPlot(pval_b_p3v3lf2, pval_s_p3v3lf2,
    byAveLogCPM = TRUE, selected.method = s.method)
```

Supplementary Figure 11v

```
# dir is your local directory
# containing Rdata file
# load(paste0(dir,
# 'Rdata/supp_sim.Rdata'))
s.method <- c("edgeR", "edgeR_robust",
    "limma_voom", "DESeq2", "DESeq_glm",
    "DESeq_cr", "EBSeq")
summPlot(pval_b_p3v3lf3, pval_s_p3v3lf3,
    byAveLogCPM = TRUE, selected.method = s.method)
```

Supplementary Figure 11vi

```
# dir is your local directory
# containing Rdata file
# load(paste0(dir,
# 'Rdata/supp_sim.Rdata'))
s.method <- c("edgeR", "edgeR_robust",
    "limma_voom", "DESeq2", "DESeq_glm",
    "DESeq_cr", "EBSeq")
summPlot(pval_b_p3v3lf6, pval_s_p3v3lf6,
    byAveLogCPM = TRUE, selected.method = s.method)
```

Supplementary Figure 12i

```
# dir is your local directory
# containing Rdata file
par(mfcol = c(1, 2))
par(mar = c(5, 5, 4, 2))
s.method <- c("edgeR", "edgeR_robust",
    "limma_voom", "DESeq2", "DESeq_pool",
    "DESeq_glm", "EBSeq")
summFold(pval_b_p10v10lF, selected.method = s.method)
mtext("(a)", side = 3, adj = -0.1,
    padj = -0.7, cex = 2.5)
summFold(pval_s_p10v10lF, selected.method = s.method)
mtext("(b)", side = 3, adj = -0.1,
    padj = -0.7, cex = 2.5)
resetPar()
```

Supplementary Figure 12ii

```
# dir is your local directory
# containing Rdata file
par(mfcol = c(1, 2))
par(mar = c(5, 5, 4, 2))
s.method <- c("edgeR", "edgeR_robust",
    "limma_voom", "DESeq2", "DESeq_pool",
    "DESeq_glm", "EBSeq")
summFold(pval_b_p3v3lF, selected.method = s.method)
mtext("(a)", side = 3, adj = -0.1,
    padj = -0.7, cex = 2.5)
summFold(pval_s_p3v3lF, selected.method = s.method)
mtext("(b)", side = 3, adj = -0.1,
    padj = -0.7, cex = 2.5)
resetPar()
```

Simulation for Supplementary Figure 9, 11 and 12

```
source("http://130.60.190.4/robinson_lab/edgeR_robust/robust_simulation.R")
method <- c("edgeR", "edgeR_robust",
    "limma_voom", "DESeq2", "DESeq_glm",
    "DESeq_cr", "DESeq_pool", "DESeq2_rmNA",
    "samr_SAMseq", "EBSeq")
s.method <- c("edgeR", "edgeR_robust",
    "edgeR_rdev", "limma_voom",
    "DESeq2", "DESeq_glm", "DESeq_cr",
    "DESeq_pool", "DESeq2_rmNA",
    "samr_SAMseq", "EBSeq")
fold_seq <- c(1, runif(5, 2, 2.2),
```

```r
    runif(5, 3, 3.3), runif(5,
        6, 6.6))
n10 <- 20
g10 <- as.factor(rep(0:1, each = n10/2))
n5 <- 10
g5 <- as.factor(rep(0:1, each = n5/2))
n3 <- 6
g3 <- as.factor(rep(0:1, each = n3/2))
######### pickrell############################################
p5v5lf2 <- NBsim(foldDiff = 2,
    dataset = pickrell, nTags = 30000,
    group = g5, add.outlier = TRUE,
    outlierMech = c("S", "R", "M"),
    pOutlier = 0.1)
pval_b_p5v5lf2 <- pval(p5v5lf2,
    method = method, count.type = "counts",
    mc.cores = 8)
pval_s_p5v5lf2 <- pval(p5v5lf2,
    method = method, count.type = "S",
    mc.cores = 8)
p5v5lf3 <- NBsim(foldDiff = 3,
    dataset = pickrell, nTags = 30000,
    group = g5, add.outlier = TRUE,
    outlierMech = c("S", "R", "M"),
    pOutlier = 0.1)
pval_b_p5v5lf3 <- pval(p5v5lf3,
    method = s.method, count.type = "counts",
    mc.cores = 8)
pval_s_p5v5lf3 <- pval(p5v5lf3,
    method = s.method, count.type = "S",
    mc.cores = 8)
p5v5lf6 <- NBsim(foldDiff = 6,
    dataset = pickrell, nTags = 30000,
    group = g5, add.outlier = TRUE,
    outlierMech = c("S", "R", "M"),
    pOutlier = 0.1)
pval_b_p5v5lf6 <- pval(p5v5lf6,
    method = method, count.type = "counts",
    mc.cores = 8)
pval_s_p5v5lf6 <- pval(p5v5lf6,
    method = method, count.type = "S",
    mc.cores = 8)
p3v3lf2 <- NBsim(foldDiff = 2,
    dataset = pickrell, nTags = 30000,
    group = g3, add.outlier = TRUE,
    outlierMech = c("S", "R", "M"),
    pOutlier = 0.1)
```

```r
pval_b_p3v3lf2 <- pval(p3v3lf2,
    method = method, count.type = "counts",
    mc.cores = 8)
pval_s_p3v3lf2 <- pval(p3v3lf2,
    method = method, count.type = "S",
    mc.cores = 8)
p3v3lf3 <- NBsim(foldDiff = 3,
    dataset = pickrell, nTags = 30000,
    group = g3, add.outlier = TRUE,
    outlierMech = c("S", "R", "M"),
    pOutlier = 0.1)
pval_b_p3v3lf3 <- pval(p3v3lf3,
    method = method, count.type = "counts",
    mc.cores = 8)
pval_s_p3v3lf3 <- pval(p3v3lf3,
    method = method, count.type = "S",
    mc.cores = 8)
p3v3lf6 <- NBsim(foldDiff = 6,
    dataset = pickrell, nTags = 30000,
    group = g3, add.outlier = TRUE,
    outlierMech = c("S", "R", "M"),
    pOutlier = 0.1)
pval_b_p3v3lf6 <- pval(p3v3lf6,
    method = method, count.type = "counts",
    mc.cores = 8)
pval_s_p3v3lf6 <- pval(p3v3lf6,
    method = method, count.type = "S",
    mc.cores = 8)
###### by different folds####
p3v3lF <- NBsimFold(fold_seq = fold_seq,
    dataset = pickrell, group = g3,
    add.outlier = TRUE, pOutlier = 0.1)
pval_b_p3v3lF <- pval(p3v3lF, method = method,
    count.type = "counts", mc.cores = 16)
pval_s_p3v3lF <- pval(p3v3lF, method = method,
    count.type = "S", mc.cores = 16)
p5v5lF <- NBsimFold(fold_seq = fold_seq,
    dataset = pickrell, group = g5,
    add.outlier = TRUE, pOutlier = 0.1)
pval_b_p5v5lF <- pval(p5v5lF, method = method,
    count.type = "counts", mc.cores = 16)
pval_s_p5v5lF <- pval(p5v5lF, method = method,
    count.type = "S", mc.cores = 16)
p10v10lF <- NBsimFold(fold_seq = fold_seq,
    dataset = pickrell, group = g10,
    add.outlier = TRUE, pOutlier = 0.1)
pval_b_p10v10lF <- pval(p10v10lF,
```

```
    method = method, count.type = "counts",
    mc.cores = 16)
pval_s_p10v10lF <- pval(p10v10lF,
    method = method, count.type = "S",
    mc.cores = 16)
######### cheung#############################################
c5v5lf3 <- NBsim(foldDiff = 3,
    dataset = cheung, nTags = 10000,
    group = g5, add.outlier = TRUE,
    outlierMech = c("S", "R", "M"),
    pOutlier = 0.1)
pval_b_c5v5lf3 <- pval(c5v5lf3,
    method = method, count.type = "counts",
    mc.cores = 8)
pval_s_c5v5lf3 <- pval(c5v5lf3,
    method = method, count.type = "S",
    mc.cores = 8)
# dir is your local directory
# containing Rdata file
save(list = c(ls(pattern = "p10v10"),
    ls(pattern = "p5v5"), ls(pattern = "p3v3"),
    ls(pattern = "c5v5")), file = paste0(dir,
    "Rdata/supp_sim.Rdata"))
```

2.2 Rcode for Supplementary Table

Supplementary Table 1

```
# dir is your local directory
# containing Rdata file
load(paste0(dir, "Rdata/manuscript.Rdata"))
library(DESeq)
# pickrell
ids_out <- c("ENSG00000158270",
    "ENSG00000125378", "ENSG00000134762")
i_out <- match(ids_out, rownames(tags))
ids <- rownames(tags)
i <- match(ids, rownames(tagsw))
tw <- tagsw[i, ]$table[, c("logFC",
    "logCPM", "LR", "PValue", "FDR")]
id_de <- match(ids, res$id)
tde <- res[id_de, ][, c("pval",
    "padj")]
id <- match(ids, rownames(d))
final_tags <- cbind(tags, dispersion = d$tagwise.dispersion[id],
    tw, dispersion = dw$tagwise.dispersion[id],
    tde, perGeneDispEsts = fitInfo(de)$perGeneDispEsts[id_de],
```

```
    fittedDispEsts = fitInfo(de)$fittedDispEsts[id_de])
# witten
ids_out_witten <- "miR-133b"
i_out_witten <- match(ids_out_witten,
    rownames(tags_witten))
ids_witten <- rownames(tags_witten)
i_witten <- match(ids_witten, rownames(tagsw_witten))
tw_witten <- tagsw_witten[i_witten,
    ]$table[, c("logFC", "logCPM",
    "LR", "PValue", "FDR")]
id_de_witten <- match(ids_witten,
    res_de_witten$id)
tde_witten <- res_de_witten[id_de_witten,
    ][, c("pval", "padj")]
id_witten <- match(ids_witten,
    rownames(d_witten))
final_tags_witten <- cbind(tags_witten,
    dispersion = d_witten$tagwise.dispersion[id_witten],
    tw_witten, dispersion = dw_witten$tagwise.dispersion[id_witten],
    tde_witten, perGeneDispEsts = fitInfo(de_witten)$perGeneDispEsts[id_de_witten],
    fittedDispEsts = fitInfo(de_witten)$fittedDispEsts[id_de_witten])
# export Excel xls file
library(xlsx)
outwb <- createWorkbook()
# Define some cell styles
# within that workbook
csSheetTitle <- CellStyle(outwb) +
    Font(outwb, heightInPoints = 14,
        isBold = TRUE)
csTableRowNames <- CellStyle(outwb) +
    Font(outwb, isBold = TRUE)
csTableColNames <- CellStyle(outwb) +
    Font(outwb, isBold = TRUE) +
    Alignment(wrapText = TRUE,
        h = "ALIGN_CENTER") + Border(color = "black",
    position = c("TOP", "BOTTOM"),
    pen = c("BORDER_THIN", "BORDER_THICK"))
T1 <- csSheetTitle + Alignment(h = "ALIGN_CENTER") +
    Fill("lightblue")
T2 <- csSheetTitle + Alignment(h = "ALIGN_CENTER") +
    Fill("lightgreen")
T3 <- csSheetTitle + Alignment(h = "ALIGN_CENTER") +
    Fill("orange")
B <- Border(color = "black", position = "LEFT",
    pen = "BORDER_THIN")
sheet <- createSheet(outwb, sheetName = "Pickrell data")
addDataFrame(final_tags, sheet,
```

```
    startRow = 2, startColumn = 1,
    colnamesStyle = csTableColNames,
    rownamesStyle = csTableRowNames)
setColumnWidth(sheet, colIndex = 1,
    colWidth = 17)
rows = createRow(sheet, 1)
createCell(rows, 1:30)
block1 <- CellBlock(sheet, 1, 12,
    52, 6, create = FALSE)
CB.setBorder(block1, B, 1:52, 1)
block2 <- CellBlock(sheet, 1, 18,
    52, 6, create = FALSE)
CB.setBorder(block2, B, 1:52, 1)
block3 <- CellBlock(sheet, 1, 24,
    52, 4, create = FALSE)
CB.setBorder(block3, B, 1:52, 1)
addMergedRegion(sheet, 1, 1, 12,
    17)
addMergedRegion(sheet, 1, 1, 18,
    23)
addMergedRegion(sheet, 1, 1, 24,
    27)
sheetTitle <- getCells(rows, colIndex = c(12,
    18, 24))
setCellValue(sheetTitle[[1]], "edgeR")
setCellValue(sheetTitle[[2]], "edgeR_robust")
setCellValue(sheetTitle[[3]], "DESeq")
setCellStyle(sheetTitle[[1]], T1)
setCellStyle(sheetTitle[[2]], T2)
setCellStyle(sheetTitle[[3]], T3)
for (i in i_out + 2) {
    cb <- CellBlock(sheet, i, 1,
        1, 27, create = FALSE)
    fill <- Fill("gray")
    CB.setFill(cb, fill, 1, 1:27)
}
sheet_witten <- createSheet(outwb,
    sheetName = "Witten data")
addDataFrame(final_tags_witten,
    sheet_witten, startRow = 2,
    startColumn = 1, colnamesStyle = csTableColNames,
    rownamesStyle = csTableRowNames)
setColumnWidth(sheet, colIndex = 1,
    colWidth = 17)
rows = createRow(sheet_witten,
    1)
createCell(rows, 1:100)
```

```r
block1 <- CellBlock(sheet_witten,
    1, 60, 52, 6, create = FALSE)
CB.setBorder(block1, B, 1:52, 1)
block2 <- CellBlock(sheet_witten,
    1, 66, 52, 6, create = FALSE)
CB.setBorder(block2, B, 1:52, 1)
block3 <- CellBlock(sheet_witten,
    1, 72, 52, 4, create = FALSE)
CB.setBorder(block3, B, 1:52, 1)
addMergedRegion(sheet_witten, 1,
    1, 60, 65)
addMergedRegion(sheet_witten, 1,
    1, 66, 71)
addMergedRegion(sheet_witten, 1,
    1, 72, 75)
sheetTitle <- getCells(rows, colIndex = c(60,
    66, 72))
setCellValue(sheetTitle[[1]], "edgeR")
setCellValue(sheetTitle[[2]], "edgeR_robust")
setCellValue(sheetTitle[[3]], "DESeq")
setCellStyle(sheetTitle[[1]], T1)
setCellStyle(sheetTitle[[2]], T2)
setCellStyle(sheetTitle[[3]], T3)
for (i in i_out_witten + 2) {
    cb <- CellBlock(sheet_witten,
        i, 1, 1, 75, create = FALSE)
    fill <- Fill("gray")
    CB.setFill(cb, fill, 1, 1:75)
}
saveWorkbook(outwb, "Supplement Table 1.xlsx")
```

Supplementary Table 2

```r
# dir is your local directory
# containing Rdata file
# load(paste0(dir,
# 'Rdata/manuscript.Rdata'))
library(xlsx)
write.xlsx(extra_table, "Supplement Table 2.xlsx",
    row.names = TRUE)
```

References

[1] Zeileis, A., Kleiber, C., and Jackman, S. (2007) *Journal Of Statistical Software* **27(8)**, 1076–84.

[2] Li, J. and Tibshirani, R. (2013) *Statistical Methods in Medical Research* **22(5)**, 519–39.
